# Facile Synthesis of Light-Switchable Polymers with Diazocine Units in the Main Chain

**DOI:** 10.3390/polym15051306

**Published:** 2023-03-05

**Authors:** Shuo Li, Katrin Bamberg, Yuzhou Lu, Frank D. Sönnichsen, Anne Staubitz

**Affiliations:** 1 University of Bremen, Institute for Organic and Analytical Chemistry, Leobener Strasse 7, D-28359 Bremen, Germany; 2 University of Bremen, MAPEX Center for Materials and Processes, Bibliothekstraße 1, D-28359 Bremen, Germany; 3 Kiel University, Otto-Diels-Institute for Organic Chemistry, Otto-Hahn-Platz 4, D-24098 Kiel, Germany

**Keywords:** photoswitch, thiol-ene, main-chain diazocine polymer, photochromism, hydrodynamic size expansion, DOSY NMR

## Abstract

Unlike azobenzene, the photoisomerization behavior of its ethylene-bridged derivative, diazocine, has hardly been explored in synthetic polymers. In this communication, linear photoresponsive poly(thioether)s containing diazocine moieties in the polymer backbone with different spacer lengths are reported. They were synthesized in thiol-ene polyadditions between a diazocine diacrylate and 1,6-hexanedithiol. The diazocine units could be reversibly photoswitched between the (*Z*)- and (*E*)-configurations with light at 405 nm and 525 nm, respectively. Based on the chemical structure of the diazocine diacrylates, the resulting polymer chains differed in their thermal relaxation kinetics and molecular weights (7.4 vs. 43 kDa) but maintained a clearly visible photoswitchability in the solid state. Gel permeation chromatography (GPC) measurements indicated a hydrodynamic size expansion of the individual polymer coils as a result of the *Z*→*E* pincer-like diazocine switching motion on a molecular scale. Our work establishes diazocine as an elongating actuator that can be used in macromolecular systems and smart materials.

## 1. Introduction

Photoswitching of molecular systems is a powerful tool to modulate their chemical and physical properties with spatiotemporal control [1]. The most prominent photoswitch, azobenzene, acts as a photochromic molecule with two interconvertible configurations, the planar C_2h_-symmetrical (*E*) isomer and the bent (*Z*) isomer in which the phenyl rings are twisted by 30° [2]. Azobenzene and its derivatives have been applied in many advanced materials [3,4], molecular machines [5] and biological systems [6]. The photoisomerization of synthetic polymers that contain azobenzene groups in the main chain leads to conformational changes of the polymer backbone, often resulting in the contraction and expansion of the polymer chains [7,8]. Particularly in polymers with semi-rigid backbones, the collective motion of azobenzene groups can induce reversible helical folding [9,10,11], show aggregation behavior for an amplified photoresponse and alter the electrochemical conductivity in π-conjugated chains [12]. Recent advances in main-chain type azobenzene-containing polymers also focused on the photocontrol of semi-crystalline and liquid crystalline properties [13,14,15]. The resulting photoinduced phase transitions enabled reversible photomelting [15,16], photomechanical actuation of thin films [17,18,19,20], as well as surface relief gratings after polarized light illumination with interference patterns [21,22].

**Scheme 1:**
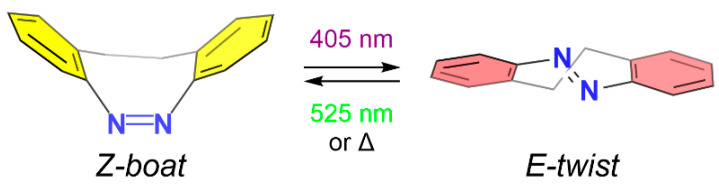
Photochemical isomerization reaction between (*Z*)- and (*E*)-diazocine.

Among azobenzene modifications, so-called diazocines enjoy special attention because the relative thermodynamical stability of their photoswitchable isomers is reversed compared to the parent azobenzene: the bent (*Z*) isomer is thermodynamically favored, and the elongated (*E*) form is metastable [23]. Synthetic procedures to obtain functionalized diazocines include the reduction of 2,2′-dinitrobibenzyl [24], oxidation of 2,2′-diaminobibenzyl [25] and the cascade amidation reaction of 2,2′-dihalobibenzyl compounds via Boc-protected diazocines [26]. The photoconversion of the yellow (*Z*) to the red (*E*) isomer occurs at an irradiation wavelength of 405 nm, resulting in an energetic increase of 37.08 kJ/mol (Scheme 1) [27]. The photochromism of diazocine is reversible with green light with a wavelength of 525 nm or via thermal relaxation with a half-life in hexane of 4.5 h at 28.5 °C [23]. According to the X-ray crystal structures of the 3,3′-diaminosubstituted diazocine provided by Sell and co-workers, the amino nitrogen atoms were further apart upon switching from *Z* (8 Å) to *E* (11 Å) configuration [28]. This molecular pincer motion was exploited to gain photocontrol over macromolecular systems such as peptides [29] and oligonucleotides [30]. Li and co-workers prepared diazocine-inserted polyurea in which photoisomerization triggered an amorphous-to-crystalline transition of the material [31]. Photomechanical thin films were produced; however, probably due to complex intermolecular interactions, no thermal phase transitions were detected. Although the photoinduced shrinking of polymers with integrated azobenzene moieties in the main chain has been studied for various kinds of polymers [13,32,33], the potential of size-switching of linear polymers containing multiple diazocine groups has not been reported yet.

In this communication, we explore the possibilities of photoinduced hydrodynamic size expansion by insertion of multiple diazocine groups into the polymer backbone as a result of their collective pincer-type switching motions. Based on our synthetic cross-coupling strategy towards substituted diazocines, we recently generated diazocines as alkyl halide initiators for their incorporation into polymeric chains via atom transfer radical polymerization [34]. Herein, we focus on diazocine diacrylates as monomers in thiol-ene polyadditions to form photochromic units in the polymer main chain with different alkyl spacer lengths. In contrast to the previously reported polyurea where the microstructure is stabilized by hydrogen bonds [31], poly(thioether)s create much weaker interactive forces and hydrogen bonds are absent. Until now, the properties and possibilities of diazocine in synthetic polymers have hardly been explored. We report on the synthesis, photochromism, thermal analysis and hydrodynamic size-switching of diazocine-containing poly(thioether)s. The expected photoinduced size expansions of the polymer coils were quantified using gel permeation chromatography (GPC) and ^1^H diffusion-ordered nuclear magnetic resonance spectroscopy (^1^H DOSY NMR).

## 2. Materials and Methods

### 2.1. Materials

Syntheses under Schlenk conditions or in a glovebox (Pure LabHE from Inert, Amesbury, MA, USA) were performed with nitrogen as the protection gas. All glassware was dried in an oven at 200 °C for at least 2 h prior to use. Syringes that were used to transfer anhydrous solvents or reagents were purged with nitrogen prior to use. Microwave reaction vials were used as sealed tubes and equipped with a septum cap from Biotage (Biotage, Uppsala, Sweden). CH_2_Cl_2_ (ACS grade, >99.9%), CHCl_3_ (p.a. 99.0–99.4%), diethyl ether (>99.8%, contained butylated hydroxytoluene), ethyl acetate (ACS grade, >99.5%) and toluene (ACS grade, >99.7%) were purchased from Sigma-Aldrich (St. Louis, MO, USA). Cyclohexane (ACS grade, >99.5%) and THF (reagent grade, >99% contained 250 ppm butylated hydroxytoluene) were purchased from Honeywell (Morristown, NJ, USA). All solvents for purification and extraction were used as received. Solvents used for synthesis under inert conditions (CH_2_Cl_2_, THF, toluene) were dried using a solvent purification system (SPS) from Inert Corporation (Amesbury, MA, USA). 1,9-Nonanediol (98%, from TCI, Tokyo, Japan) and acryloyl chloride (96%, stabilized with 400 ppm phenothiazine, from Alfa Aesar, Ward Hill, MA, USA), Na_2_SO_4_ (ACS grade, 99.0%, from Merck, Darmstadt, Germany), NaCl (>99%, from T.H. Geyer, Renningen, Germany), NaHCO_3_ (analytical reagent grade, from Fisher Scientific, Pittsburgh, PA, USA), NaOH (pellets, from VWR, Radnor, PA, USA), NH_4_Cl (>99.7% p.a., from Roth, Karlsruhe, Germany), pyridine (99.5%, from Grüssing, Filsum, Germany) and thionyl chloride (99.7%, from Fisher Scientific, Pittsburgh, USA) were used as received. 1,6-Hexanedithiol (HDT, 97+%, from Apollo, Cheshire, UK), dimethylphenylphosphine (DMPP, 97%, from Alfa Aesar, Ward Hill, MA, USA), dimethylformamide (DMF, 99.8%, extra dry, from Fisher Scientific, Pittsburgh, PA, USA) and triethylamine (TEA, anhydrous, from Fluorochem, Hadfield, UK) were stored in the glovebox. Spin-coating was performed with polymer solutions (3 mg/mL) on Menzel-Gläser coverslips (Thermo Fischer Scientific, Waltham, MA, USA) (18 mm × 18 mm) at 150 rps for 1 min.

### 2.2. Methods

NMR spectra were recorded on a Bruker Avance Neo 600 (Bruker BioSpin, Rheinstetten, Germany) (600 MHz (^1^H), 151 MHz (^13^C{^1^H})) at 298 K. ^1^H DOSY NMR spectra were recorded on a Bruker Avance II HD 600 (Bruker BioSpin, Rheinstetten, Germany) (600 MHz (^1^H)) at 298 K with 32 increments, 8 scans, 14 ppm spectral width, 2.5 s delay time and 130 ms diffusion delay time and analyzed with MestReNova 11.0.4 (Metrelab Research, Santiago de Compostela, Spain) and Bruker TopSpin 4.0.6 (Bruker Biospin, Rheinstetten, Germany) software. All ^1^H NMR and ^13^C{^1^H} NMR spectra were referenced to the residual proton signals of the solvent (^1^H) or the solvent itself (^13^C{^1^H}). The exact assignment of the peaks was performed using two-dimensional NMR spectroscopy such as ^1^H,^1^H-COSY; ^1^H,^13^C-HSQC; and ^1^H,^13^C-HMBC when possible. Photostationary states (PSS) of compounds **M1**, **M2**, **P1**, **P2** were determined using ^1^H NMR spectroscopy (1 mM in for monomers **M1** and **M2**, 3 mg/mL for polymers **P1** and **P2** in THF-*d*_8_) at 25 °C. Compound irradiations were performed directly on sample solutions in the NMR tubes with light at 405 nm or 525 nm wavelength for 2 min before the NMR spectra were recorded.

High-resolution EI mass spectra were recorded on a MAT 95XL double-focusing mass spectrometer from Finnigan MAT (Thermo Fisher Scientific, Waltham, MA, USA) at an ionization energy of 70 eV. Samples were measured using a direct or indirect inlet method with a source temperature of 200 °C. High-resolution ESI and APCI mass spectra were measured using a direct inlet method on an Impact II mass spectrometer from Bruker (Bruker Daltonics, Bremen, Germany). ESI mass spectra were recorded in the positive ion collection mode.

IR spectra were recorded on a Nicolet i510 FT-IR spectrometer from Thermo Fisher Scientific (Thermo Fisher Scientific, Waltham, MA, USA) with a diamond window in an area from 500 to 4000 cm^−1^ with a resolution of 4 cm^−1^. All samples were measured 16 times against a background scan.

Melting points were recorded on a Büchi Melting Point M-560 (Büchi, Essen, Germany) and are reported corrected.

Thin layer chromatography (TLC) was performed using TLC Silica gel 60 F254 from Merck (Merck, Darmstadt, Germany) and compounds were visualized using exposure to UV light at a wavelength of 254 nm. Column chromatography was performed by using SiO_2_ (0.040–0.063 mm, 230–400 mesh ASTM) from Merck.

Irradiation experiments were carried out using LED light sources of 405 nm central wavelength (optical power = 680 mW; intensity = 2.2 mW/cm^2^) and 525 nm central wavelength (optical power = 20 W; intensity = 64 mW/cm^2^) at a 2 cm distance from the object.

UV-vis absorption measurements were recorded on a Perkin Elmer UV/VIS NIR Spectrometer Lambda (PerkinElmer, Waltham, MA, USA) 900 at 298 K. Quartz cuvettes of 10 mm optical path length were used. The absorption maxima at wavelengths λ_max_ and thermal relaxation kinetics were determined using UV-vis spectroscopy (1 mM in for monomers **M1** and **M2** in THF, 0.5 mg/mL for polymers **P1** and **P2** in THF) at 25 °C. The cuvettes and the spin-coated films were irradiated with light at 405 nm or 525 nm wavelength for 2 min before the absorption spectra were measured. The thermal relaxation kinetics were recorded three times using UV-vis spectroscopy (1 mM in for monomers **M1** and **M2** in THF, 3 mg/mL for polymers **P1** and **P2** in THF) at 25 °C. The cuvettes were irradiated with light at 405 nm wavelength for 2 min before 37 spectra were recorded in the dark in 5 min intervals. The absorption at λ_max_(*E*) was plotted against the reaction time before the rate constant k and the half-life *t*_1/2_ were determined via first-order reaction kinetics.

Diffusion coefficients *D* of compounds **M1**, **M2**, **P1**, **P2** were determined using ^1^H DOSY NMR spectroscopy (1 mM for monomers **M1** and **M2** in THF-*d*_8_, 3 mg/mL for polymers **P1** and **P2** in THF-*d*_8_) at 25 °C under the same irradiation conditions as above. The average over the three aromatic diazocine signals and the resulting standard error were determined. Due to the fast relaxation kinetics of the (*E*)-diazocines in **M1** and **P1**, the DOSY signal intensities were corrected and normalized. For the (*Z*)-isomer, a time offset *f* was necessary to describe the hypothetical time at which I(*Z*) = 1:

1 − (I(*Z*)/I_0_(*Z*)) = e^−k (t+*f*)^ (1)

This time *f* was calculated with the following equation:

I(*Z*) = (amount of *E* at t = 0) × e^−k *f*^ = 1 (2)

In addition to the experimental time of the DOSY, the period between irradiation and the start of the DOSY experiment must also be considered. The time of each data point of the DOSY results from the quotient of the experimental time and the number of intervals between the data points, which is successively added to the start time of the DOSY after the irradiation. The corrected and normalized intensity I′ results from the following equation:

I′ = *n* × I_0_ (3)

The normalization factor *n* was chosen so that the intensities of the first data point, both in the measured and in the corrected data set, stay identical. These corrected and normalized intensities were plotted as a function of the gradient strength *G* and fitted with the Stejskal–Tanner equation [35]:

I′ = e^(−*D* × 4*π*^2^*γ*^2^*δ*^2^*G*^2^((∆ − *δ*)/2)) × I_0_ (4)

*D*: diffusion coefficient in cm^2^ s^−1^, *γ*: gyromagnetic ratio, *δ*: pulse width, ∆: diffusion delay time, *G*: gradient strength. The hydrodynamic radii were determined using the Stokes–Einstein equation:

r = k × T/(6π × η × *D*) (5)

with k: Boltzmann constant, T: temperature, and *D*: diffusion coefficient. The dynamic viscosity η of THF-*d*_8_ at 298 K was adopted from Dowds and co-workers [32]:

η = 4.84 × 10^−4^ Pa×s (6)

Gel permeation chromatography (GPC) was performed using a PSS (polymer standard service) SECurity GPC system with a conventional calibration using polystyrene standards. The polymers were dissolved in THF (1 mg/mL) and the GPC elugrams were recorded at an elution flow rate of 1 mL/min. Molecular weights *M*_n_ and *M*_w_ were obtained from the molar mass distribution via GPC analysis using PSS WinGPC^®^ UniChrom 8.20 (PSS GmbH, Mainz, Germany) software. Apparent molecular weights of the polymers **P1** and **P2** (1 mg/mL in THF) were calculated from molar mass distributions using GPC at 35 °C. The open vials were irradiated from above with light at 405 nm or 525 nm wavelength for 2 min and capped before the polymer solution was injected into the GPC system. The dispersity *Đ* was calculated from GPC data and is defined as the ratio between weight average (*M*_w_) and number average molar mass (*M*_n_):

*Đ* = *M*_w_/*M*_n_ (7)

Differential scanning calorimetry measurements (DSC) were performed on a Mettler Toledo DSC3+ instrument in aluminum crucibles (100 µL) at a heating rate of 10 K/min under N_2_ with a flow rate of 20 mL/min. The first heating and cooling curves were used. Glass transition temperatures *T*_g_ of compounds **P1** (sample weight: 6.96 mg) and **P2** (sample weight: 8.79 mg) were determined as the inflection points between onset and endpoint temperatures of the DSC plot. The polymer-coated aluminum crucibles were irradiated from above with light at 405 nm or 525 nm wavelength for 2 min before the DSC plots were recorded in the dark.

### 2.3. Synthetic Procedures

**9-Hydroxynonyl acrylate**


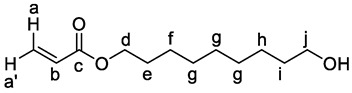


A dry, nitrogen-flushed Schlenk-flask equipped with a magnetic stirring bar and a septum was charged with nonane-1,9-diol (3.60 g, 22.4 mmol), anhydrous THF (25 mL) and triethylamine (3.2 mL, 22.08 mmol), and the flask was cooled to 0 °C prior to the dropwise addition of acryloyl chloride (1.66 mL, 20.4 mmol) over a course of 30 min. The reaction mixture was stirred at 0 °C for 4 h before allowing the reaction mixture to warm slowly to 20 °C, at which temperature it was stirred for a further 12 h. The reaction mixture was quenched with saturated aq NaHCO_3_ (30 mL), extracted with ethyl acetate (3 × 30 mL), washed with brine (30 mL) and dried over Na_2_SO_4_. After filtration, the organic phase was concentrated under reduced pressure and the crude residue was purified using silica gel column chromatography (cyclohexane/ethyl acetate 50/50) to furnish the product **9-hydroxynonyl acrylate** as a colorless oil (1.107 g, 5.17 mmol, 23%).

**^1^H NMR (601 MHz, CDCl_3_):** *δ* = 6.39 (dd, *J* = 17.3, 1.5 Hz, 1H, H-a), 6.11 (dd, *J* = 17.3, 10.4 Hz, 1H, H-b), 5.81 (dd, *J* = 10.4, 1.5 Hz, 1H, H-a′), 4.14 (t, *J* = 6.7 Hz, 2H, H-d), 3.63 (t, *J* = 6.6 Hz, 2H, H-j), 1.70–1.62 (m, 2H, H-e), 1.60–1.52 (m, 2H, H-i), 1.38–1.28 (m, 10H, H-f, H-g, H-h) ppm.

**^13^C{^1^H} NMR (151 MHz, CDCl_3_):** *δ* = 166.5 (C-c), 130.6 (C-a), 128.8 (C-b), 64.8 (C-d), 63.2 (C-j), 32.9 (C-i), 29.6 (C-g), 29.4 (C-g), 29.3 (C-g), 28.7 (C-e), 26.0 (C-f), 25.8 (C-h) ppm.

**HRMS (ESI)** *m/z* for C_12_H_22_O_3_ [M + H]^+^: calcd 215.16417, found: 215.16415.

**IR (ATR):** *ṽ* = 3351 (w), 2927 (m), 2855 (m), 1723 (s), 1636 (w), 1465 (w), 1408 (m), 1295 (m), 1272 (m), 1188 (s), 1056 (w), 983 (m), 810 (m), 754 (s) cm^−1^.

***R_f_*:** 0.30 (cyclohexane/ethyl acetate = 80/20)

**Bis(9-(acryloyloxy)nonyl) (*Z*)-11,12-dihydrodibenzo[*c*,*g*][1,2]diazocine-2,9-dicarboxylate (M1)**

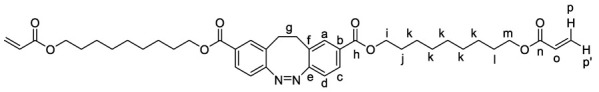



A nitrogen-flushed Schlenk-flask equipped with a magnetic stirring bar and a reflux condenser was charged with compound **1** (325 mg, 1.10 mmol, reference [26]) and thionyl chloride (3.2 mL, 44.00 mmol). The reaction mixture was stirred under nitrogen at 76 °C for 3 h before it was cooled to 20 °C. Then, low-boiling compounds were removed using vacuum distillation (55 °C, 100 mbar) and the solid residue was washed with dry DCM (2 × 5 mL) before it was stored under a nitrogen atmosphere.

In a glovebox, a sealed tube was charged with the solid residue, dry toluene (11 mL), pyridine (370 µL, 4.53 mmol) and 9-hydroxynonyl acrylate (551 mg, 2.57 mmol). The vial was capped, transferred out of the glovebox and stirred at 100 °C for 3 h. After cooling to 20 °C, the reaction mixture was quenched with water (50 mL), extracted with ethyl acetate (3 × 30 mL), washed with saturated aq NH_4_Cl (30 mL) and brine (30 mL), and dried over Na_2_SO_4_. After filtration, the organic phase was concentrated under reduced pressure and the crude residue was purified using silica gel column chromatography (cyclohexane to cyclohexane/ethyl acetate 75/25) to furnish the product **M1** as a yellow solid (481 mg, 700 µmol, 63%).

**^1^H NMR (601 MHz, CDCl_3_):** *δ* = 7.79 (dd, *J* = 8.2, 1.7 Hz, 2H, H-c), 7.67 (d, *J* = 1.7 Hz, 2H, H-a), 6.88 (d, *J* = 8.2 Hz, 2H, H-d), 6.39 (dd, *J* = 17.3, 1.5 Hz, 2H, H-p), 6.11 (dd, *J* = 17.3, 10.4 Hz, 2H, H-o), 5.81 (dd, *J* = 10.4, 1.5 Hz, 2H, H-p′), 4.23 (td, *J* = 6.8, 2.2 Hz, 4H, H-i), 4.14 (t, *J* = 6.7 Hz, 4H, H-m), 3.06–2.84 (m, 4H, H-g), 1.75–1.67 (m, 4H, H-l), 1.68–1.63 (m, 4H, H-j), 1.45–1.28 (m, 20H, H-k) ppm.

**^1^H NMR (600 MHz, THF):** *δ* = 7.77 (dd, *J* = 8.2, 1.7 Hz, 2H), 7.70 (d, *J* = 1.7 Hz, 2H), 6.88 (d, *J* = 8.2 Hz, 2H), 6.31 (dd, *J* = 17.3, 1.7 Hz, 2H), 6.10 (dd, *J* = 17.3, 10.4 Hz, 2H), 5.78 (dd, *J* = 10.4, 1.7 Hz, 2H), 4.19 (qt, *J* = 10.9, 6.7 Hz, 4H), 4.10 (t, *J* = 6.7 Hz, 4H), 3.01–2.91 (m, 4H), 1.71–1.67 (m, 4H), 1.64 (p, *J* = 6.7 Hz, 4H), 1.46–1.26 (m, 20H) ppm.

**^13^C{^1^H} NMR (151 MHz, CDCl_3_):** *δ* = 166.5 (C-n), 165.8 (C-h), 158.8 (C-e), 131.5 (C-a), 130.6 (C-p), 129.5 (C-b), 128.8 (C-o), 128.5 (C-c), 128.2 (C-f), 118.7 (C-d), 65.5 (C-i), 64.8 (C-m), 31.4 (C-g), 29.5 (C-k), 29.3 (C-k), 28.8 (C-12), 28.7 (C-j), 26.1 (C-k), 26.0 (C-k) ppm.

**HRMS (ESI)** *m/z* for C_40_H_53_N_2_O_8_ [M + H]^+^: calcd 689.37964, found: 689.37890.

**IR (ATR):** *ṽ* = 2930 (w), 2853 (w), 1712 (s), 1633 (w), 1476 (s), 1408 (m), 1276 (m), 1252 (m), 1190 (s), 1134 (m), 1012 (w), 959 (m), 892 (w), 809 (m), 758 (m), 723 (w) cm^−1^.

**mp:** 58 °C.

***R_f_*:** 0.52 (cyclohexane/ethyl acetate = 80/20)

**(*Z*)-(11,12-dihydrodibenzo[*c*,*g*][1,2]diazocine-2,9-diyl)bis(methylene) diacrylate (M2)** 
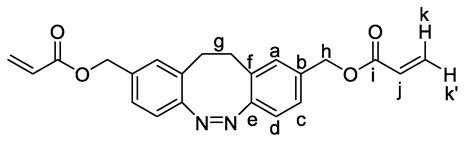


In a glovebox, compound **2** (268 mg, 1.00 mmol, reference [26]), dry DMF (5 mL) and dry TEA (550 μL, 4.00 mmol) were added into a sealed tube. The tube was capped with a crimp cap equipped with a PTFE septum, transferred out of the glovebox and cooled down to 0 °C. Acryloyl chloride (200 μL, 2.00 mmol) was dissolved in dry DMF (2.5 mL) and added dropwise within 5 min. The mixture was stirred at 0 °C for 1 h, then warmed up to 20 °C and stirred for a further 24 h. The solution was diluted and extracted with DCM (50 mL), washed with H_2_O (2 × 15 mL) and brine (10 mL), and dried over Na_2_SO_4_. After filtration, the organic phase was concentrated under reduced pressure and the crude residue was purified using silica gel column chromatography (cyclohexane/ethyl acetate 70/30) to furnish product **M2** as a yellow solid (140 mg, 370 µmol, 37%).

**^1^H NMR (601 MHz, CDCl_3_):** *δ* = 7.16 (dd, *J* = 8.1, 1.8 Hz, 2H, H-c), 7.00 (d, *J* = 1.8 Hz, 2H, H-a), 6.86 (d, *J* = 8.1 Hz, 2H, H-d), 6.43 (dd, *J* = 17.3, 1.3 Hz, 2H, H-k), 6.14 (dd, *J* = 17.3, 10.5 Hz, 2H, H-j), 5.85 (dd, *J* = 10.4, 1.3 Hz, 2H, H-k′), 5.06 (s, 4H, H-h), 2.88 (m, 4H, H-g) ppm.

**^1^H NMR (600 MHz, THF):** *δ* = 7.16 (dd, *J* = 8.0, 1.6 Hz, 2H), 7.05 (d, *J* = 1.4 Hz, 2H), 6.79 (d, *J* = 8.0 Hz, 2H), 6.34 (dd, *J* = 17.3, 1.6 Hz, 2H), 6.12 (dd, *J* = 17.3, 10.4 Hz, 2H), 5.81 (dd, *J* = 10.4, 1.6 Hz, 2H), 5.02 (d, *J* = 8.9 Hz, 4H), 2.97–2.76 (m, 4H) ppm.

**^13^C{^1^H} NMR (151 MHz, CDCl_3_):** *δ* = 166.0 (C-i), 155.3 (C-e), 134.9 (C-b), 131.5 (C-k), 129.6 (C-a), 128.3 (C-f, C-j), 126.8 (C-c), 119.5 (C-d), 65.7 (C-h), 31.8 (C-g) ppm.

**HRMS (EI)** *m/z* for C_22_H_20_N_2_O_4_ [M]^+^: calcd 376.14231, found: 376.14203 (30); 55 (100).

**IR (ATR):** *ṽ* = 2961 (w), 1718 (s), 1633 (w), 1404 (s), 1294 (m), 1265 (m), 1174 (s), 1046 (m), 967 (m), 807 (s), 735 (w), 657 (w) cm^−1^.

**mp:** 62 °C.

***R_f_*:** 0.55 (cyclohexane/ethyl acetate = 67/33)

**Poly[3,3′-hexane-1,6-diylbis(sulfanediyl) bis(propionyloxynonyl) (*Z*)-(11,12-dihydrodibenzo[*c*,*g*][1,2]diazocine-2,9-diacarboxylate] (P1)**

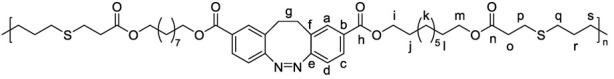



In a glovebox, a sealed tube was charged with compound **M1** (400 mg, 580 µmol), THF (1 mL) and 1,6-hexanedithiol (84.0 µL, 550 µmol). Dimethylphenylphosphine (0.80 µL, 5.80 µmol) was added, the tube was capped with a crimp cap equipped with a PTFE septum and the reaction mixture was stirred at 20 °C for 40 min. Then, the tube was transferred out of the glovebox and the reaction mixture was precipitated dropwise in methanol (5 mL). The resulting solid was collected, redissolved in THF (1 mL) and re-precipitated in methanol (10 mL). The resulting solid was collected, redissolved in THF (1 mL) and re-precipitated in diethyl ether (10 mL) before the solid residue was dried in vacuum (50 °C, 48 h) to furnish the product **P1** (322 mg, 67%) as a yellow solid.

**^1^H NMR (600 MHz, THF):** *δ* = 7.77 (dd, *J* = 8.2, 1.7 Hz, 2H, H-c), 7.70 (d, *J* = 1.7 Hz, 2H, H-a), 6.89 (d, *J* = 8.2 Hz, 2H, H-d), 4.28–4.14 (m, 4H, H-i), 4.12–4.01 (m, 4H, H-m), 3.05–2.90 (m, 4H, H-g), 2.79–2.68 (m, 4H, H-p), 2.54 (t, *J* = 7.4 Hz, 4H, H-o), 2.51 (t, *J* = 7.4 Hz, 4H, H-q), 1.72–1.65 (m, 4H, H-j), 1.65–1.53 (m, 8H, H-l, H-r), 1.45–1.37 (m, 8H, H-k, H-s), 1.37–1.30 (m, 16H, H-k) ppm.

**^13^C{^1^H} NMR (151 MHz, THF):***δ* = 172.2 (C-n), 165.9 (C-h), 160.3 (C-e), 132.2 (C-a), 130.4 (C-b), 129.5 (C-f), 129.1 (C-c), 119.5 (C-d), 65.8 (C-i), 65.1 (C-m), 35.8 (C-o), 32.7 (C-q), 32.1 (C-g), 30.8 (C-k), 30.6 (C-k), 30.5 (C-k), 30.3 (C-k), 30.3, (C-l) 29.8 (C-j), 29.5 (C-s), 27.9 (C-p), 27.1 (C-k), 27.0 (C-k) ppm.

**IR (ATR):** *ṽ* = 2918 (m), 2850 (m), 1715 (s), 1605 (w), 1463 (m), 1347 (m), 1285 (s), 1247 (s), 1163 (s), 1110 (s), 1000 (m), 961 (m), 756 (s), 722 (w) cm^−1^.

**Poly[(*Z*)-(11,12-dihydrodibenzo[*c*,*g*][1,2]diazocine-2,9-dimethyl-3,3′-(hexane-1,6-diylbis(sulfanediyl))dipropionate] (P2)**

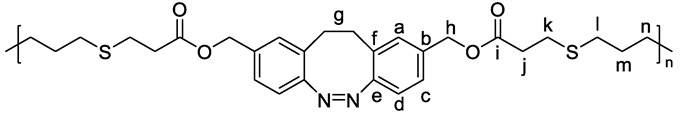



In a glovebox, a sealed tube was charged with compound **M2** (217 mg, 580 µmol), THF (1 mL) and 1,6-hexanedithiol (84.0 µL, 550 µmol). Dimethylphenylphosphine (0.80 µL, 5.80 µmol) was added, the tube was capped with a crimp cap equipped with a PTFE septum and the reaction mixture was stirred at 20 °C for 40 min. Then, the tube was transferred out of the glovebox and reaction mixture was precipitated dropwise in methanol (5 mL). The resulting solid was collected, redissolved in THF (2.5 mL) and re-precipitated in methanol (10 mL). The resulting solid was collected, redissolved in THF (2.5 mL) and re-precipitated in diethyl ether (10 mL) before the solid residue was dried in vacuum (50 °C, 48 h) to furnish the product **P2** (168 mg, 56%) as a yellow solid.

**^1^H NMR (600 MHz, THF):** *δ* = 7.19–7.10 (m, 2H, H-c), 7.08–7.01 (m, 2H, H-a), 6.84–6.76 (m, 2H, H-d), 5.03–4.90 (m, 4H, H-h), 2.98–2.75 (m, 4H, H-g), 2.75–2.68 (m, 4H, H-k), 2.56 (m, 4H, H-j), 2.53–2.46 (m, 4H, H-l), 1.62–1.48 (m, 4H, H-m), 1.48–1.34 (m, 4H, H-n) ppm.

**^13^C{^1^H} NMR (151 MHz, THF):***δ* = 172.1 (C-i), 156.6 (C-e), 136.3 (C-b), 130.4 (C-a), 129.1 (C-f), 127.5 (C-c), 120.0 (C-d), 66.2 (C-h), 35.8 (C-j), 32.7 (C-l), 32.5 (C-g), 30.6 (C-m), 29.5 (C-n), 27.8 (C-k) ppm.

**IR (ATR):** *ṽ* = 2924 (m), 2851 (w), 1729 (s), 1414 (w), 1376 (w), 1343 (m), 1239 (s), 1143 (s), 962 (m), 891 (m), 827 (m), 803 (m), 734 (m), 672 (m) cm^−1^.

## 3. Results and Discussion

**Scheme 2:**
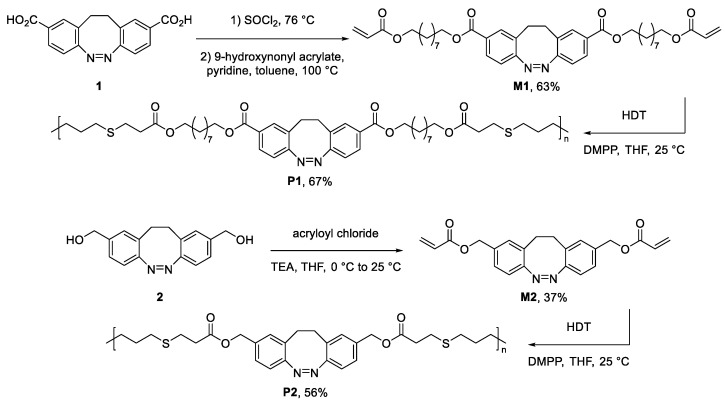
Synthesis of the monomers and polymerization. Compounds **1** and **2** were synthesized according to previously published procedures [26].

Previous work enabled the access to functionalized diazocine compounds [26], among which carboxylic acid (**1**) and dihydroxymethyl (**2**) compounds (Scheme 2) were easily formed from methyl ester as the common starting material. Acylation of the dihydroxymethyl derivative of diazocine **2** with acryloyl chloride provided the short diazocine diacrylate **M2** in a 37% yield. For the synthesis of the diacrylate comprising nonanyl alkyl spacers **M1**, the carboxylic acid functions in **1** were activated to reactive acyl chlorides before esterification with 9-hydroxynonyl acrylate established product **M1** in an overall 63% yield. Finally, the monomers **M1** and **M2** were subjected to the Michael-type thiol-ene polyaddition reaction [36] with 1,6-hexanedithiol (HDT) as the nucleophile in a 1.05:1 stoichiometric ratio to ensure acrylate groups at the polymer chain ends and to prevent disulfide links. By employing 1 mol% of dimethylphenylphosphine (DMPP) as the catalyst, the desired polymers **P1** and **P2** were formed in good yields (67% and 56%, respectively) after purification by precipitation.

**Figure 1:**
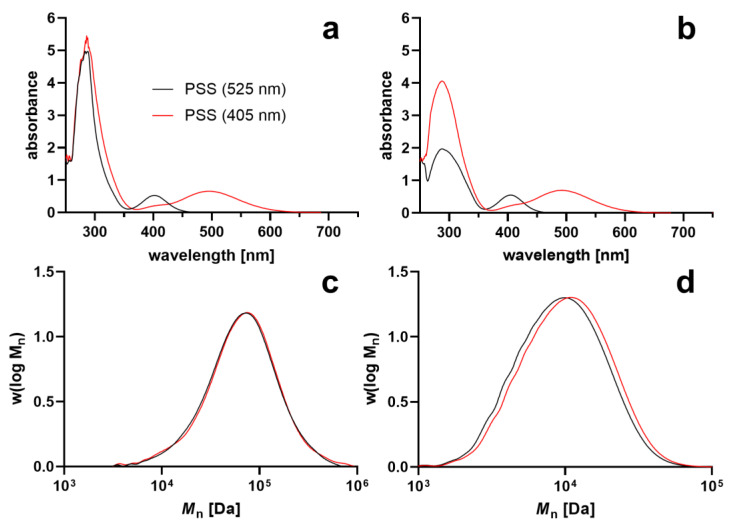
UV-vis spectra of polymers **P1** (**a**) and **P2** (**b**) at PSS (405 nm, red) and PSS (525 nm, black) at a concentration of 0.5 mg/mL in THF; and molar mass distribution of polymers **P1** (**c**) and **P2** (**d**).

**Figure 2:**
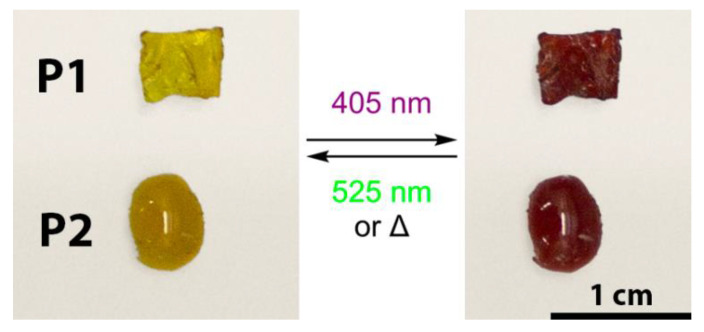
Polymer samples of **P1** and **P2** at PSS (525 nm) and PSS (405 nm).

The photochromism of the diazocine monomers and polymers was investigated using UV-vis spectroscopy at 25 °C (Table 1). The n–π* transition maxima of the (*Z*) and (*E*) isomers were detected at around 400 and 492 nm, in agreement with existing diazocine compounds (Figure 1a,b and Figure S1). No signs of photodegradation were observed for the polymers **P1** and **P2** after ten cyclic irradiation measurements (Figure S2). The spontaneous *E*→*Z* thermal relaxation of the diazocine units followed first-order reaction kinetics (Figures S3 and S4). Since the relaxation rates are predominantly influenced by the electronic effects of the proximal substituents on the aromatic rings of diazocines [25], a large difference in thermal half-lives was determined between the polymers **P1** and **P2** (39 min and 350 min) but without significant differences compared to the respective monomers **M1** and **M2** (42 min and 358 min). In the solid state, the polymers **P1** and **P2** underwent photochromism in bulk, as evident from the color change between the yellow (*Z*) and red (*E*) configurations (Figure 2). To compare the photochromism of the polymers **P1** and **P2** in the solid state with the dissolved samples, thin films were produced using spin-coating of the polymer solutions on glass slides. The photostationary states at 405 and 525 nm light irradiation wavelengths were equally reached within 1 min as no spectral changes could be detected after longer irradiation times. The absorption spectra of the thin films resemble the polymer solutions in THF, thereby showing equal photochromic efficiencies in the solid state (Figure S5).

The diazocine products were further analyzed using ^1^H NMR spectroscopy, initially showing the (*Z*) isomer in **M1**, **M2**, **P1** and **P2** (see the SI). For the polymers **P1** and **P2**, depletion of the acrylate signals of the monomer and the emergence of broad alkyl signals confirmed the incorporation of 1,6-hexanedithiol and thus the success of the thiol-ene step-growth polymerization. Furthermore, the ^1^H NMR spectrum of polymer **P2** contained end-group acrylate signals which allowed the determination of a DP of 17 using the integration of the signals between the acrylate protons and aromatic protons of the diazocine repeating units. Upon photoexcitation of the (*Z*) isomers at a wavelength of 405 nm, the photostationary states (PSS) of all diazocine products were reached within 1 min with an accumulation of the downfield-shifted (*E*) isomer in good photoconversion yields (Γ*_Z_*→*_E_*) for **M1** (60%) and **M2** (64%) (Table 1). In comparison to the monomers **M1** and **M2**, the *E*/*Z* ratios of the respective polymers **P1** and **P2** remained constant, thereby showing no signs of restraint with regard to the switching ability of the integrated diazocine units in the polymer chain. Complete conversion (Γ*_E_*→*_Z_* > 99%) to the (*Z*) isomers was reached within 1 min of light irradiation at 525 nm wavelength for all diazocine compounds **M1**, **M2**, **P1** and **P2**.

To analyze the molecular weights of the polymers, the molecular mass distributions were determined using gel permeation chromatography (GPC) in THF (Figure 1c,d). In the case of polymer **P1**, long polymer chains were obtained, with a number average molecular weight (*M*_n_) of 43 kDa and a dispersity (*Đ*) of 2.5, corresponding to a degree of polymerization (DP) of 51 (Table 2). Polymer **P2** had much shorter polymer chains than **P1** with an *M*_n_ of 7.4 kDa and a dispersity of 1.6, corresponding to a DP of 14. Since the GPC was calibrated from polystyrene standards, the DP of 17 obtained from the NMR integral measurement was expected to be more accurate. After irradiation of polymer **P2** with light at 405 nm wavelength, the apparent *M*_n_ measured using GPC increased to 8.1 kDa. In comparison to the measurement under ambient conditions, the apparent increase in *M*_n_ by 0.7 kDa reflects the collective photoisomerization of the main-chain diazocine groups which caused hydrodynamic size expansion of the individual polymer coils in THF. However, no change in the apparent *M*_n_ could be detected after light irradiation at 405 nm wavelength for **P1**, possibly due to the fast *E*→*Z* thermal relaxation of the diazocine units during the GPC elution process or because the change is small.

To study the thermal properties and potential transitions of the polymer materials **P1** and **P2**, the photoinduced enthalpic changes of the polymers **P1** and **P2** were measured as a function of temperature using differential scanning calorimetry (DSC). The long alkyl spacers between the diazocine units in polymer **P1** led to a low glass transition temperature (*T*_g_) of −11.3 °C under ambient conditions (Figures S6 and S7). The shorter polymer **P2** exhibited a *T*_g_ at 12.3 °C without further phase transitions at higher temperatures. Apart from the glass transitions, no other thermal phase transitions could be detected. After irradiation of the polymer samples with light at 405 nm wavelength, the changes in *T*_g_ as a result of the *Z*→*E* photoisomerization of the diazocine groups were negligible (0.4−2 °C). Therefore, the configurations of diazocine did not have a strong impact on the rigidity of the polymer chains and did not suggest a higher crystalline order upon photoinduced *Z*→*E* switching. In comparison to the amorphous-to-crystalline transition in diazocine-containing polyurea proposed by Li and co-workers [31], the poly(thioether) chains **P1** and **P2** did not form an intermolecular network and allowed an isotropic movement of the diazocine units. The polymers remained in a soft, rubbery state at room temperature (25 °C) due to the low *T*_g_ of **P1** and **P2**. Sufficient free volume in the polymer matrix provided a similar photochromic behavior compared to the polymer solutions in THF.

^1^H DOSY NMR spectroscopy provided useful information about the size of individual polymer coils. For polymers containing molecular switches that undergo substantial geometric changes such as the diazocine, the collective isomerization of the switching units is expected to lead to an alteration of the measured diffusion coefficients (*D*). The hydrodynamic radius (r) of an individual polymer coil is inversely proportional to the diffusion coefficient and dependent on the dynamic viscosity of the solvent given by the Stokes–Einstein equation (Equation 9). In order to detect the hydrodynamic size expansion of the polymer coils in solution, DOSY experiments at PSS of 405 and 525 nm light irradiation wavelengths were conducted in THF-*d*_8__,_ focusing on the aromatic signals of diazocine. The dynamic viscosity of THF-*d*_8_ at 25 °C was adopted from previous work by Dowds and co-workers (Equation 10) [32]. As expected at PSS (525 nm), single diffusion coefficients of the diazocine products **M1**, **M2**, **P1** and **P2** in (*Z*) configuration were obtained (for **M2** and **P2**, see the SI).

The (*E*) isomers of **M1** and in **P1** relaxed noticeably during the DOSY measurements, as the experiment duration (~20 min) was of the order of the compound half-lives (~25 min), see also Figure S8 The associated increase or decrease in concentration of (*Z*) and (*E*) species, respectively, influenced the integrals of the DOSY peaks, which in turn affected the fit quality and led to erroneous diffusion coefficients *D* for both species. In order to correct this error in the measured data, the *E*→*Z* thermal relaxation kinetics must be taken into account. Therefore, the intensities were corrected and normalized with a normalization factor *n*, and a time offset *f* was included in the calculation of the (*Z*) isomer intensities (Table S1). Finally, the corrected and normalized intensities I′ were plotted as a function of the gradient strength G and fitted with the Stejskal–Tanner equation (Figure S9) [35]. A detailed procedure can be found in the *Methods* section.

Photoswitching of the diazocine **M1** with 405 nm violet light gave rise to a new and slightly decreased diffusion coefficient of 7.39 ± 0.07 × 10^−6^ cm^2^ s^−1^ that corresponded to the (*E*) isomer compared to the steady (*Z*) isomer at 7.45 ± 0.14 × 10^−6^ cm^2^ s^−1^. In addition, the DOSY spectra of the polymers **P1** and **P2** reflected the molar mass distributions obtained using GPC analysis: Firstly, the diffusion coefficients showed higher statistical deviation attributed to the polydispersity of the polymers (see Table S1). Secondly, the higher *M*_n_ of **P1** compared to **P2** was confirmed by the lower diffusion coefficient of **P1** (1.96 ± 0.05 × 10^−6^ cm^2^ s^−1^) compared to **P2** (2.84 ± 0.08 × 10^−6^ cm^2^ s^−1^). Since each individual polymer chain in **P1** and **P2** contains multiple diazocine units, photoswitching with light at 405 nm wavelength resulted in a distribution of the (*Z*)- and (*E*)-diazocine isomers within a macromolecule. As a consequence, the diffusion behavior of the polymers in THF-*d*_8_ upon photoswitching to PSS (405 nm) is expected to shift collectively for both isomeric forms. In this case, the mean value of the diffusion coefficient of **P1** decreased from 1.96 ± 0.05 × 10^−6^ to 1.91 ± 0.02 × 10^−6^ (*Z*) and 1.90 ± 0.03 × 10^−6^ (*E*) cm^2^ s^−1^, and the diffusion coefficient of **P2** decreased from 2.84 ± 0.08 × 10^−6^ cm^2^ s^−1^ to 2.72 ± 0.21 × 10^−6^ (*Z*) and 2.75 ± 0.07 × 10^−6^ (*E*) cm^2^ s^−1^, which corresponded to an increase in hydrodynamic radius from 2.30 nm (*Z*) to about 2.36 nm (*E*), and from 1.59 nm (*Z*) to about 1.64 nm (*E*), respectively. The observed changes in size, however, are small compared to the experimental error of the DOSY NMR measurements and the distribution of the diffusion coefficients resulting from the polymer dispersity. Because of these error margins, DOSY experiments are not by themselves sufficient to prove a size change. While the photoswitching of the diazocine units is expected to alter the conformation of the polymer backbone, the overall size of the polymer coil remained largely unaffected. This was presumably caused by the conformational freedom of the interconnecting alkyl chain linkers that simultaneously ensured a non-restrained photoswitching of the diazocine units.

## 4. Conclusions

Based on our synthetic strategy for the synthesis of functionalized diazocines, diacrylates with two different alkyl spacer lengths were generated and employed as monomers in the thiol-ene polyaddition reaction with 1,6-hexanedithiol. The resulting linear polymers with diazocine moieties in the polymer backbone differed in molecular weight and glass transition temperature while closely mimicking the photochromic behavior of the respective monomers. Owing to the *Z*→*E* pincer-like motion of the diazocine switch, the flexible polymer coils experienced a photoinduced hydrodynamic size expansion of the polymer with shorter alkyl chain linkers as confirmed by an increase in the apparent molecular weight from analytical GPC measurements in THF. However, the diffusion coefficients obtained from ^1^H DOSY NMR only decreased minimally at PSS (405 nm), probably due to the conformational freedom of the interconnecting alkyl chain linkers. Our results clearly show the potential of diazocine with its favorable properties as a photoswitch in the main chain of polymers. The reactive acrylate end groups of the polymers can be further exploited in post-polymerization modifications and cross-linking reactions. Future work includes the implementation and incorporation of diazocines in more complex polymeric architectures and higher crystalline environments such as micelles, where photoinduced size-switching can be instrumentalized for targeted drug delivery [37].

## Figures and Tables

**Table 1:** Diazocine photoswitching data obtained from UV-vis and NMR spectroscopy.

	PSS (405 nm)			PSS (525 nm)				
	Γ*_Z_*→*_E_* (%) *E*	λ_max_(*E*) (nm)	*D* (10^−6^ cm^2^ s^−1^) ^1^	Γ*_E_*→*_Z_* (%) *Z*	λ_max_(Z) (nm)	*D* (10^−6^ cm^2^ s^−1^)^1^	k (*E*→*Z*) (10^−3^ min^−1^)	*t*_1/2_ (min)
**M1**	60%	491	7.45 ± 0.14 (*Z*) 7.39 ± 0.07 (*E*)	>99%	399	7.46 ± 0.11	26.73 ± 0.84	26 ± 1
**P1**	60%	496	1.90 ± 0.03 (*Z*) 1.91 ± 0.02 (*E*)	>99%	402	1.96 ± 0.05	28.56 ± 0.37	24 ± 0
**M2**	64%	488	10.4 ± 0.16 (*Z*) 11.0 ± 0.26 (*E*)	>99%	402	10.6 ± 0.09	1.90 ± 0.04	366 ± 7
**P2**	64%	492	2.72 ± 0.21 (*Z*) 2.75 ± 0.07 (*E*)	>99%	405	2.84 ± 0.08	1.90 ± 0.05	366 ± 9

^1^ After application of correction factors and Stejskal–Tanner fitting.

**Table 2:** GPC and DSC data for polymers **P1** and **P2**.

		*M*_n,GPC_ (kDa)	*Đ*	*T*_g_ (°C)
**P1**	**PSS (525 nm)**	43	2.5	−11.3
	**PSS (405 nm)**	43	2.5	−10.9
**P2**	**PSS (525 nm)**	7.4	1.6	12.3
	**PSS (405 nm)**	8.1	1.6	10.3

## Data Availability

The raw/processed data are available upon reasonable request.

## Supplementary Materials

The following supporting information can be downloaded at: https://www.mdpi.com/article/10.3390/polym15051306/s1, Figure S1: UV-vis spectra of compounds M1 (left) and M2 (right) after light irradiation at 405 nm (red) and 525 nm wavelength (black) at a concentration of 1 mM in THF; Figure S2. Cyclic UV-vis measurements of polymers P1 (left) and P2 (right) after light irradiation at 405 nm (red) and 525 nm wavelength (black) at a concentration of 0.5 mg/mL in THF; Figure S3. First-order thermal relaxation kinetics of M1 (left) and M2 (right) from PSS (405 nm wavelength) at λ_max_(*E*) at a concentration of 3 mg/mL in THF; Figure S4. First-order thermal relaxation kinetics of P1 (left) and P2 (right) from PSS (405 nm wavelength) at λ_max_(*E*) at a concentration of 3 mg/mL in THF; Figure S5. UV-vis spectra of polymers P1 (left) and P2 (right) after light irradiation at 405 (red) and 525 nm wavelength (black) as spin-coated thin films; Figure S6. DSC plots of polymer P1 at PSS (525 nm) (black) and PSS (405 nm) (red) indicating the glass transition temperature *T*_g_. The DSC measurements were cycled between −70 and 150 °C and −40 and 40 °C, respectively; Figure S7. DSC plots of polymer P2 at PSS (525 nm) (black) and PSS (405 nm) (red) indicating the glass transition temperature *T*_g_. The DSC measurements were cycled between −70 and 150 °C and −40 and 40 °C, respectively; Figure S8. Stacked plot of the 1D ^1^H NMR spectra of M1 from 8.5 to −0.5 ppm at T = 298 K. Bottom: before irradiation, center: after irradiation at t = 0 min and top: after irradiation and the DOSY experiment at t = 27.3 min; Figure S9. Example graphs are presented to highlight the effect of data intensity correction of one peak of the (*E*) (top) and (*Z*) isomer (bottom) after light irradiation at 405 nm wavelength of M1. Fit statistics are shown for the individual fits of these resonances; Figure S10. Stacked plot of the 1D ^1^H NMR spectra of polymer P1 from 8.5 to −0.5 ppm at T = 298 K. Bottom: before irradiation, center: after irradiation at t = 0 min and top: after irradiation and the DOSY experiment at t = 26.5 min; Table S1. *C*orrected diffusion coefficients *D* [10^−6^ cm^2^ s^−1^] of the (*Z*) and (*E*) isomers of M1, P1, M2, P2 after light irradiation at 405 and 525 nm wavelength from ^1^H DOSY NMR measurements.; ^1^H and ^13^C{^1^H} NMR spectra of the products.; ^1^H DOSY NMR spectra of M2 and P2. Ref [38] is cited in Supplementary Materials.
