# Supplementary material for: Facile Synthesis of Light-Switchable Polymers with Diazocine Units in the Main Chain
Source: Polymers (Basel). 2023 Mar 5;15(5):1306. doi: 10.3390/polym15051306 (PMC10007058; doi:10.3390/polym15051306)

polymers

MDPI

Supporting Information for

Facile Synthesis of a Light Switchable Polymers with Diazocine Units in the Main Chain

- Experimental Data -

Shuo Li [1,2], Katrin Bamberg [3], Yuzhou Lu [1,2], Frank D. Sönnichsen [3] and Anne Staubitz [1,2,*]

[1]University of Bremen, Institute for Organic and Analytical Chemistry Leobener Str. 7, 28359 Bremen, Germany
[2]University of Bremen, Center for Materials and Processes, MAPEX Bibliothekstr. 1, 28359 Bremen, Germany
[3]Kiel University, Otto-Diels-Institute for Organic Chemistry, Otto-Hahn-Platz 4, D-24098 Kiel, Germany
*staubitz@uni-bremen.de

Table of Contents

UV-Vis Spectra of the Products 3

DSC Plots of Polymers P1 and P2 5

^{1}H DOSY NMR Correction Factors and Fitting Graphs 6

^{1}H and $^{13}C\{^{1}H\}$ NMR Spectra of the Products 9

^{1}H DOSY NMR Spectra of M2 and P2 18

The use of abbreviations follows the conventions from the ACS Style guide [1]:

1. *The ACS Style Guide: Effective Communication of Scientific Information*; Coghill, A.M., Garson, L.R., American Chemical Society, Eds.; 3rd ed.; American Chemical Society ; Oxford University Press: Washington, DC : Oxford ; New York, 2006; ISBN 9780841239999 9780841274006.

UV-Vis Spectra of the Products

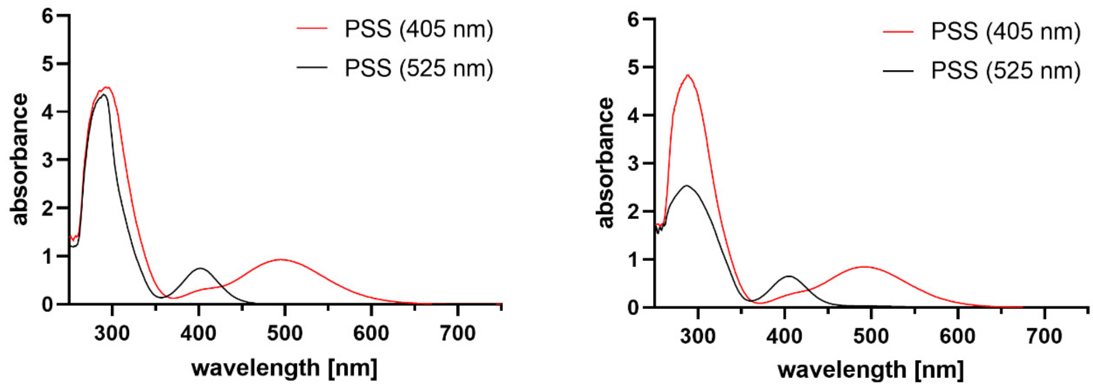

Figure S1. UV-vis spectra of compounds **M1** (left) and **M2** (right) after light irradiation at 405 nm (red) and 525 nm wavelength (black) at a concentration of 1 mM in THF.

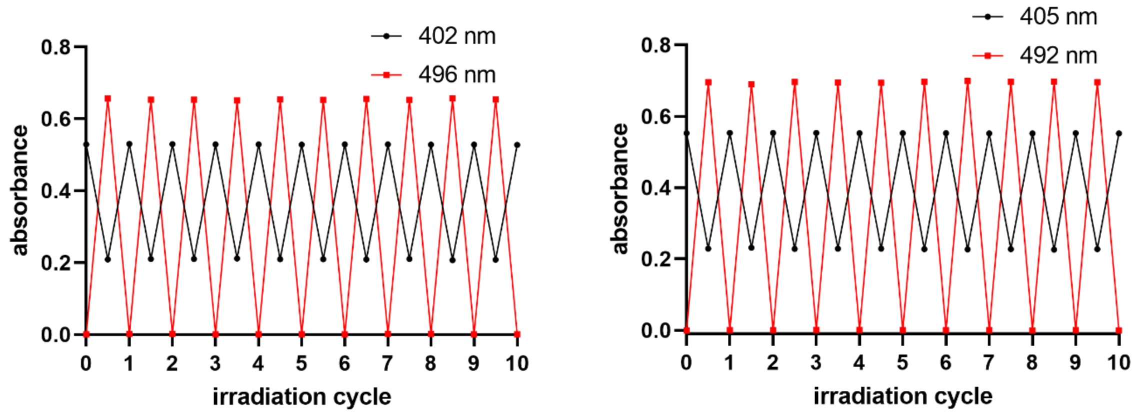

Figure S2. Cyclic UV-vis measurements of polymers **P1** (left) and **P2** (right) after light irradiation at 405 nm and 525 nm wavelength monitoring the absorption at $\lambda_{max}(Z)$ (black) and $\lambda_{max}(E)$ (red) at a concentration of 0.5 mg/mL in THF.

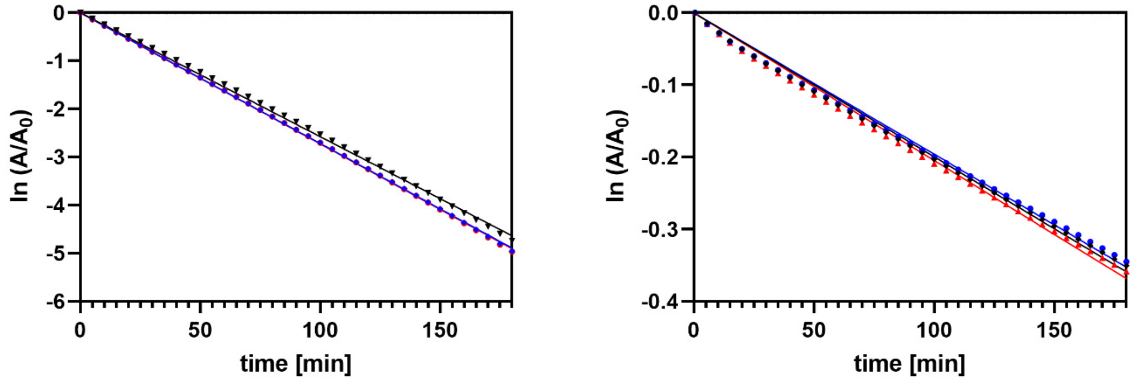

Figure S3. First-order thermal relaxation kinetics of **M1** (left) and **M2** (right) from PSS (405 nm) wavelength at $\lambda_{max}(E)$ at a concentration of 3 mg/mL in THF.

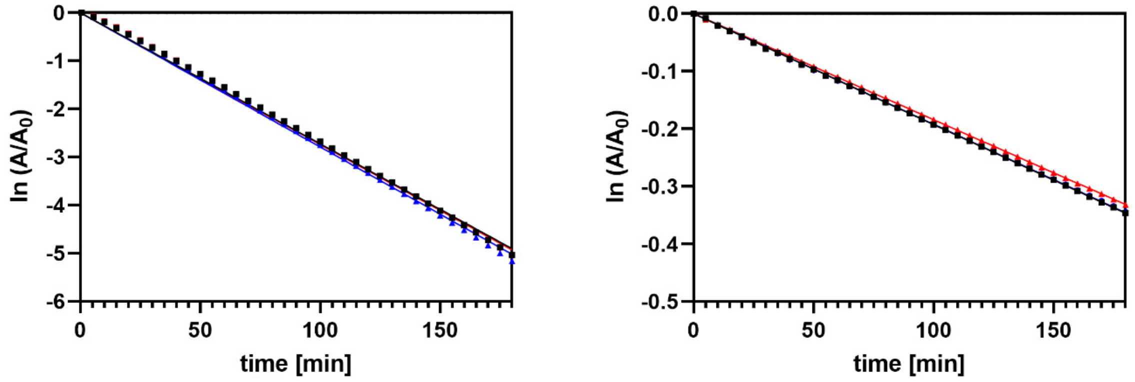

Figure S4. First-order thermal relaxation kinetics of **P1** (left) and **P2** (right) from PSS (405 nm) wavelength at $\lambda_{max}(E)$ at a concentration of 3 mg/mL in THF.

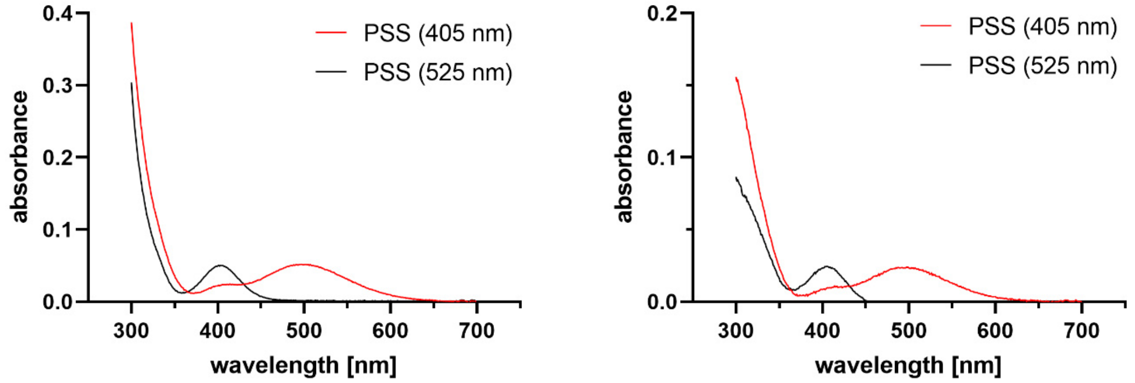

Figure S5. UV-vis spectra of polymers **P1** (left) and **P2** (right) after light irradiation at 405 (red) and 525 nm wavelength (black) as spin-coated thin films.

DSC Plots of Polymers P1 and P2

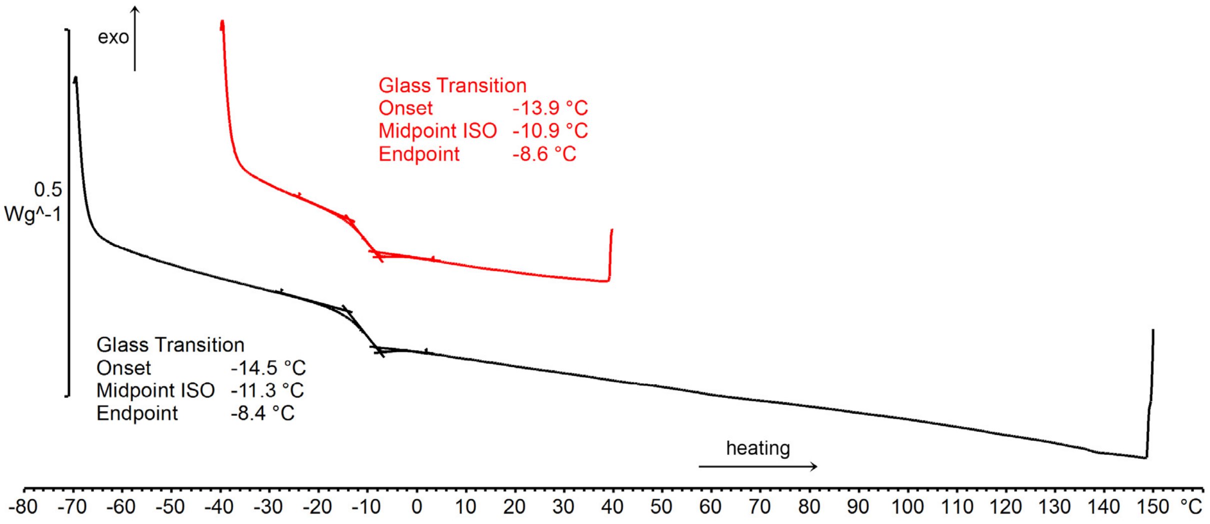

Figure S6. DSC plots of polymer **P1** at PSS (525 nm) (black) and PSS (405 nm) (red) indicating the glass transition temperature T_g. The DSC measurements were cycled between -70 to 150 ° and -40 to 40 °C, respectively.

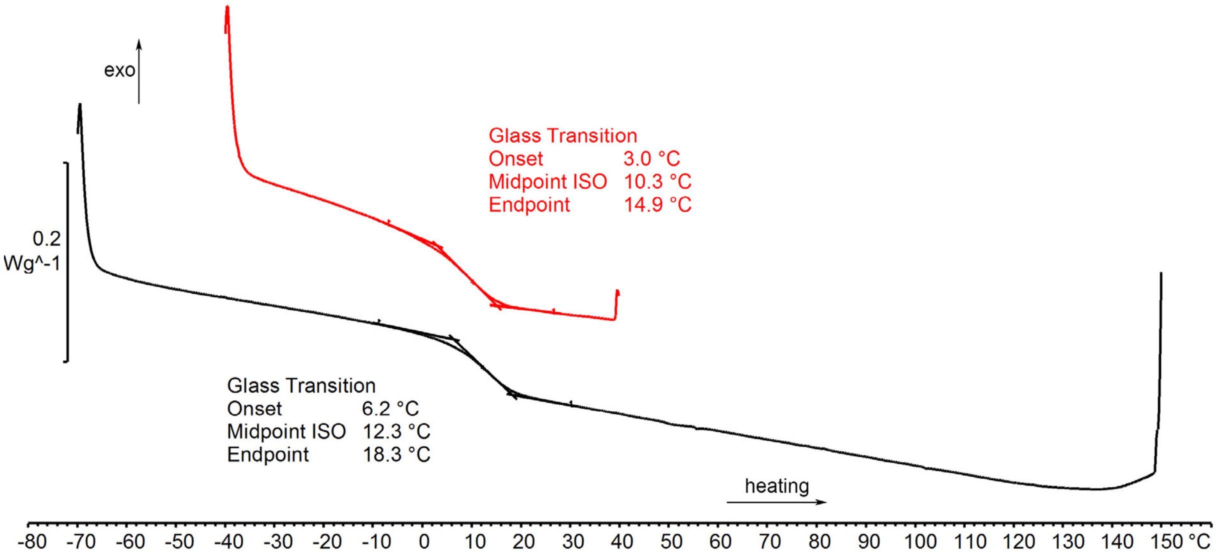

Figure S7. DSC plots of polymer **P2** at PSS (525 nm) (black) and PSS (405 nm) (red) indicating the glass transition temperature Tg. The DSC measurements were cycled between and -70 to 150 °C and -40 to 40 °C, respectively.

^{1}H DOSY NMR Correction Factors and Fitting Graphs

Bis(9-(acryloyloxy)nonyl) (*Z*)-11,12-dihydrodibenzo[*c*,*g*][1,2]diazocine-2,9-dicarboxylate (M1)

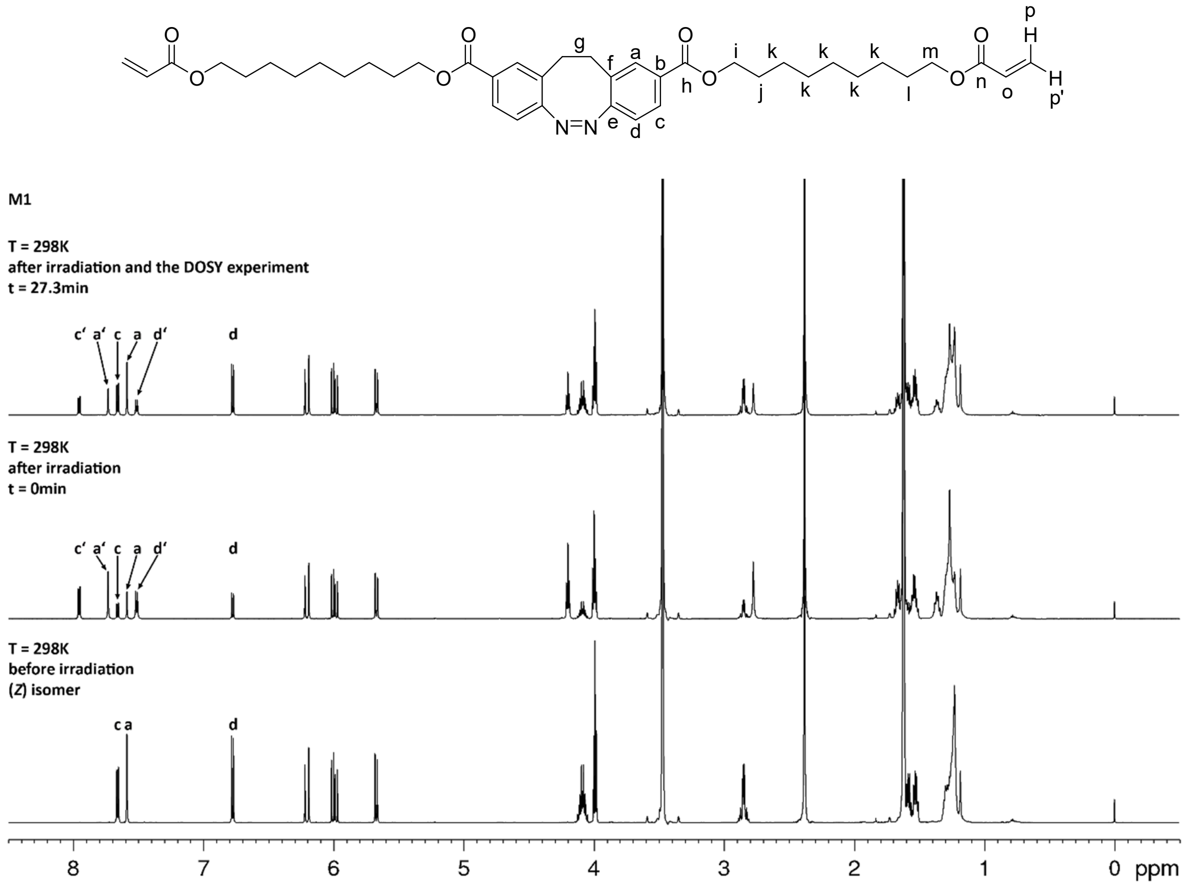

Figure S8. Stacked plot of the 1D ^{1}H NMR spectra of **M1** from 8.5 to -0.5 ppm at T = 298 K. Bottom: before irradiation, centre: after irradiation at t = 0 min and top: after irradiation and the DOSY experiment at t = 27.3 min.

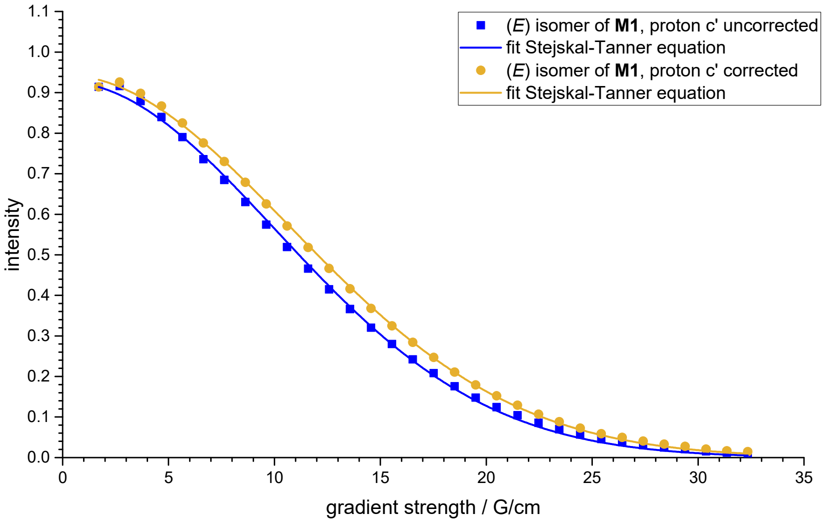

Model	Stejskal-Tanner
Equation	I*exp(-D*(4*pi*pi*gamma*gamma*LD*LD*x*x)*(BD-LD/3)*1E4)
Plot	uncorrected
D	8.27715E-10
I	0.92718
Reduced Chi-Sqr	8.26333E-5
R-Square(COD)	0.9992
Adj. R-Square	0.99917

Model	Stejskal-Tanner
Equation	I*exp(-D*(4*pi*pi*gamma*gamma*LD*LD*x*x)*(BD-LD/3)*1E4)
Plot	corrected and normalized
D	7.3177E-10
I	0.94363
Reduced Chi-Sqr	3.56541E-5
R-Square(COD)	0.99967
Adj. R-Square	0.99966

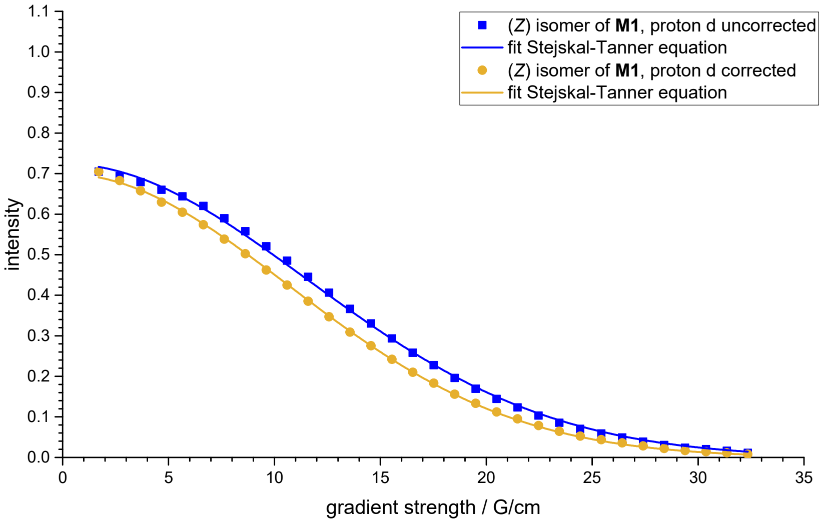

Model	Stejskal-Tanner
Equation	I*exp(-D*(4*pi*pi*gamma*gamma*LD*LD*x*x)*(BD-LD/3)*1E4)
Plot	uncorrected
D	6.27137E-10
I	0.72438
Reduced Chi-Sqr	4.14913E-5
R-Square(COD)	0.99934
Adj. R-Square	0.99932

Model	Stejskal-Tanner
Equation	I*exp(-D*(4*pi*pi*gamma*gamma*LD*LD*x*x)*(BD-LD/3)*1E4)
Plot	corrected and normalized
D	7.33443E-10
I	0.69948
Reduced Chi-Sqr	1.33843E-5
R-Square(COD)	0.99977
Adj. R-Square	0.99977

Figure S9. Example graphs are presented to highlight the effect of data intensity correction of one peak of the (E) (top) and (Z) isomer (bottom) after light irradiation at 405 nm wavelength of **M1**. Fit statistics are shown for the individual fits of these resonances.

Poly[3,3′-hexane-1,6-diylbis(sulfanediyl) bis(propionyloxynonyl) (*Z*)-(11,12-dihydrodibenzo[*c*,*g*][1,2]diazocine-2,9-diacarboxylate] (P1)

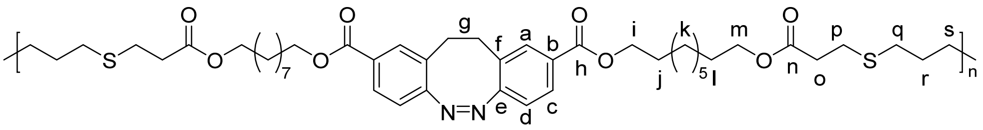

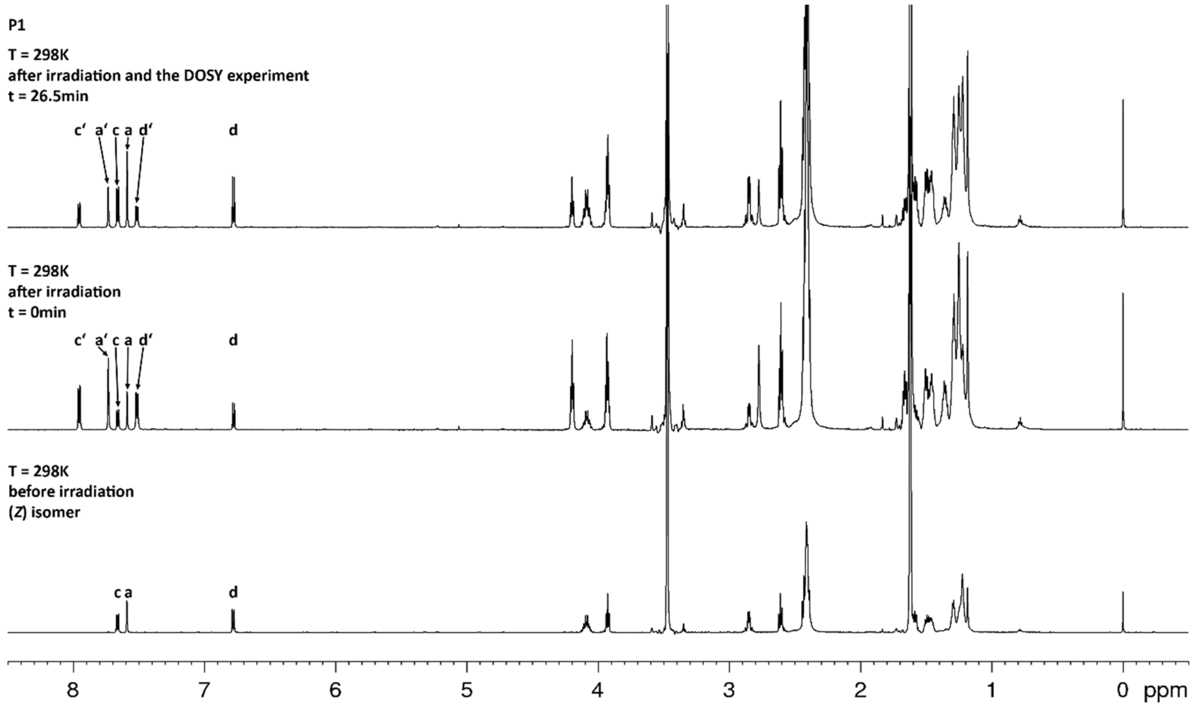

Figure S10. Stacked plot of the 1D ^{1}H NMR spectra of polymer **P1** from 8.5 to -0.5 ppm at T = 298 K. Bottom: before irradiation, centre: after irradiation at t = 0 min and top: after irradiation and the DOSY experiment at t = 26.5 min.

Aromatic proton assignments of **M1**, **P1**, **M2**, **P2** for ^{1}H DOSY NMR:

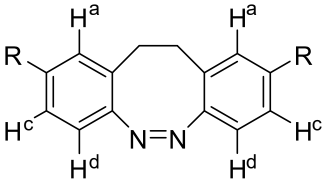

Table S1. Corrected diffusion coefficients D [10^{-6} cm^2 s^{-1}] of the (Z) and (E) isomers of **M1**, **P1**, **M2**, **P2** after light irradiation at 405 and 525 nm wavelength from ^{1}H DOSY NMR measurements.

		**PSS (405 nm)**			**PSS (525 nm)**	
	***Z* (average)**	***Z* (c; a; d)**	***E* (average)**	*E* (c'; a'; d')	*Z* (average)	*Z* (c; a; d)
		7.60±0.04		7.32±0.04		7.58±0.03
**M1**[1]	7.45±0.14	7.43±0.05	7.39±0.07	7.41±0.06	7.46±0.11	7.42±0.02
		7.33±0.04		7.45±0.03		7.37±0.03
		1.90±0.03		1.89±0.03		2.00±0.03
**P1**[2]	1.90±0.03	1.87±0.03	1.91±0.02	1.93±0.03	1.96±0.05	1.91±0.03
		1.92±0.03		1.91±0.03		1.98±0.03
		10.3±0.26		11.3±0.04		10.7±0.05
**M2**	10.4±0.16	10.3±0.10	11.0±0.26	10.8±0.04	10.6±0.09	10.5±0.04
		10.6±0.06		10.8±0.11		10.7±0.06
		2.91±0.03		2.79±0.03		2.84±0.02
**P2**	2.72±0.21	2.74±0.03	2.75±0.07	2.79±0.03	2.84±0.08	2.93±0.03
		2.49±0.02		2.67±0.03		2.76±0.03

[1]Amount of (*E*) at t = 0: 68.5%; f = 22.93 min. [2]Amount of (*E*) at t = 0: 67.2%; f = 17.20 min.

^{1}H and ^{13}C{^{1}H} NMR Spectra of the Products

9-Hydroxynonyl acrylate

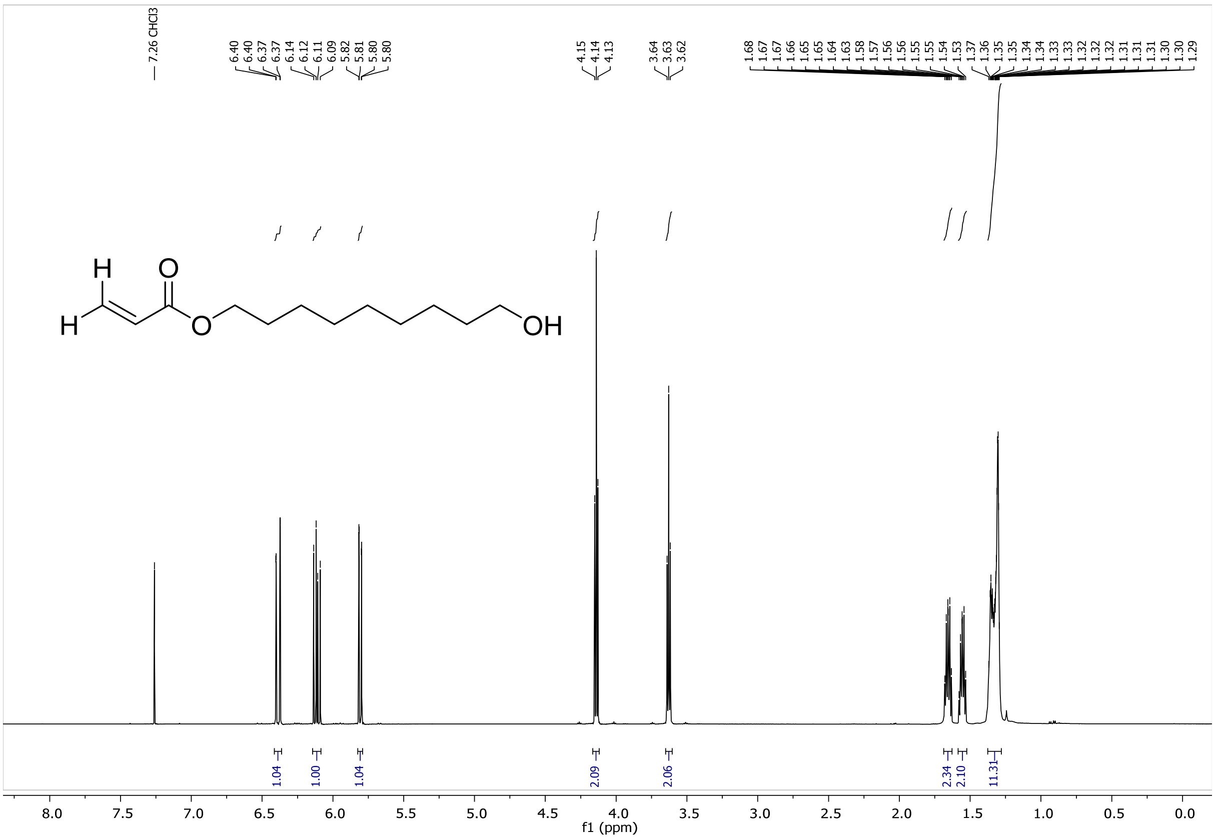

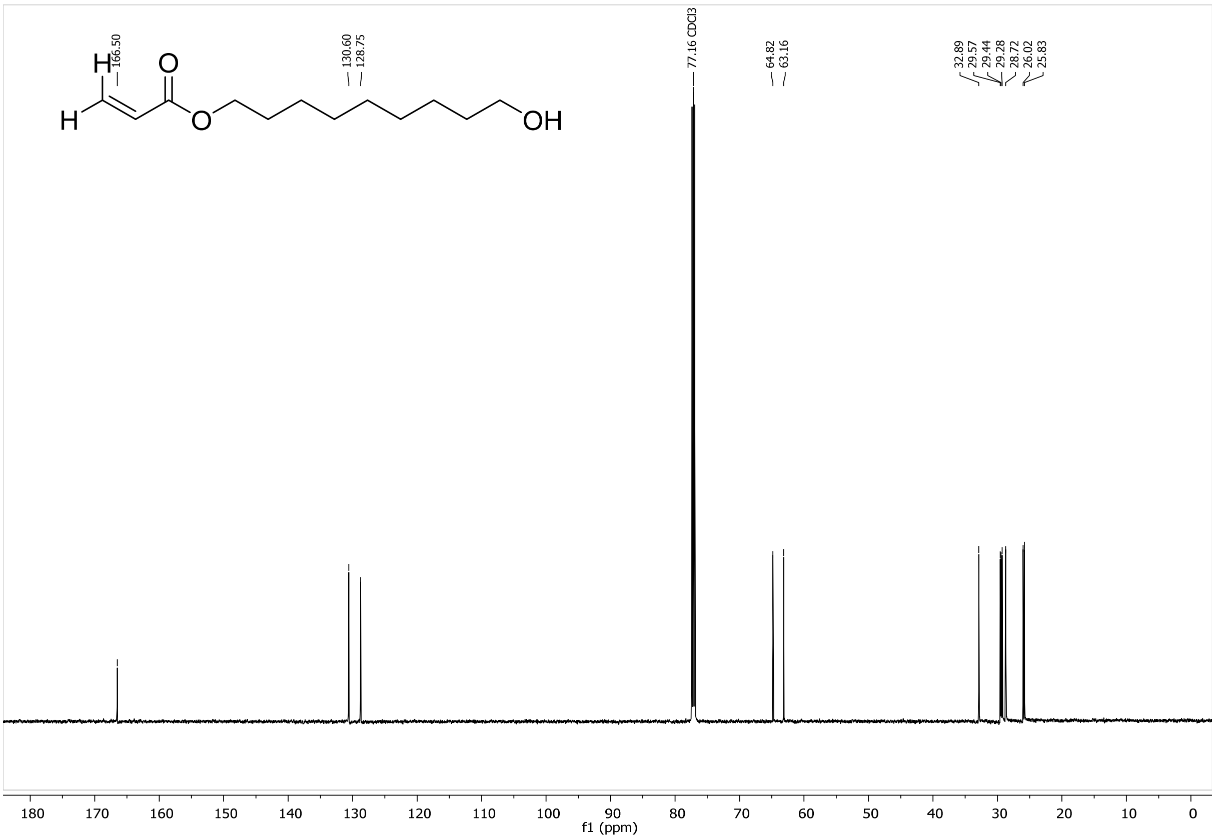

Bis(9-(acryloyloxy)nonyl) (*Z*)-11,12-dihydrodibenzo[*c*,*g*][1,2]diazocine-2,9-dicarboxylate (M1)

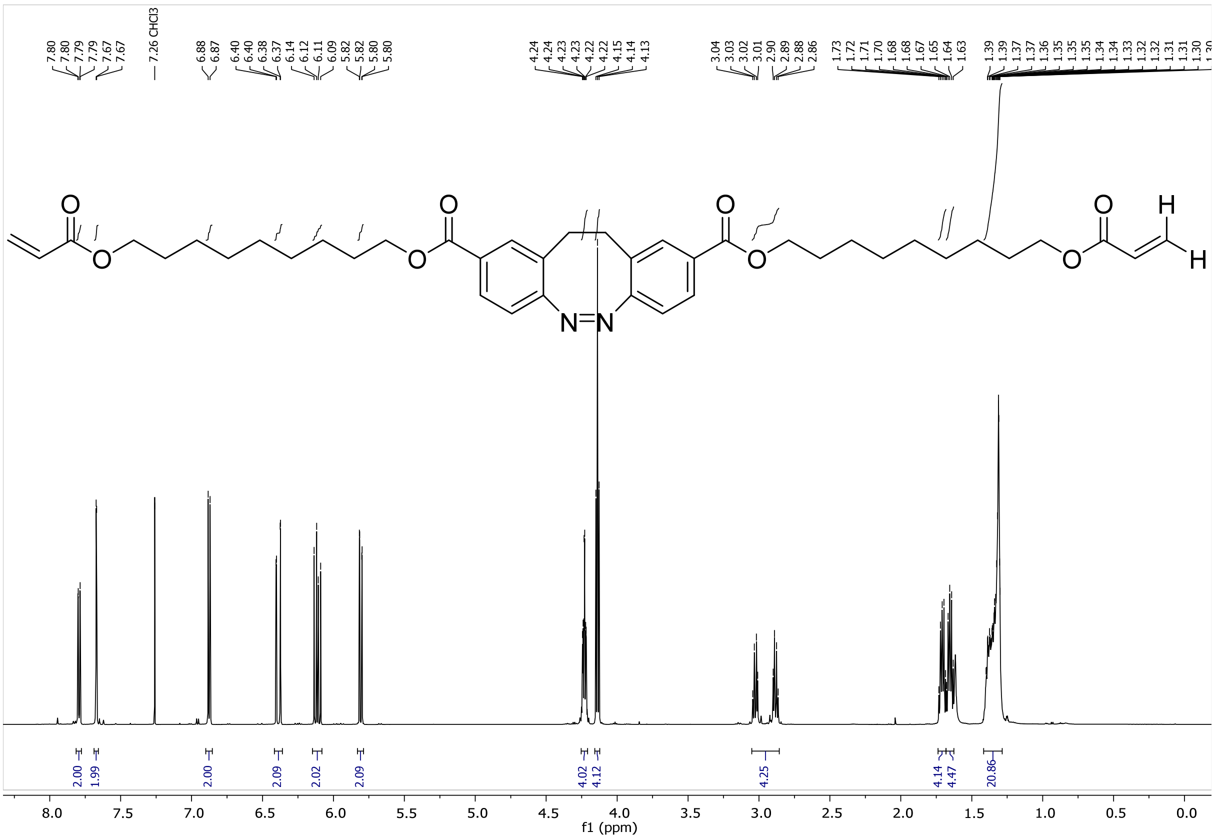

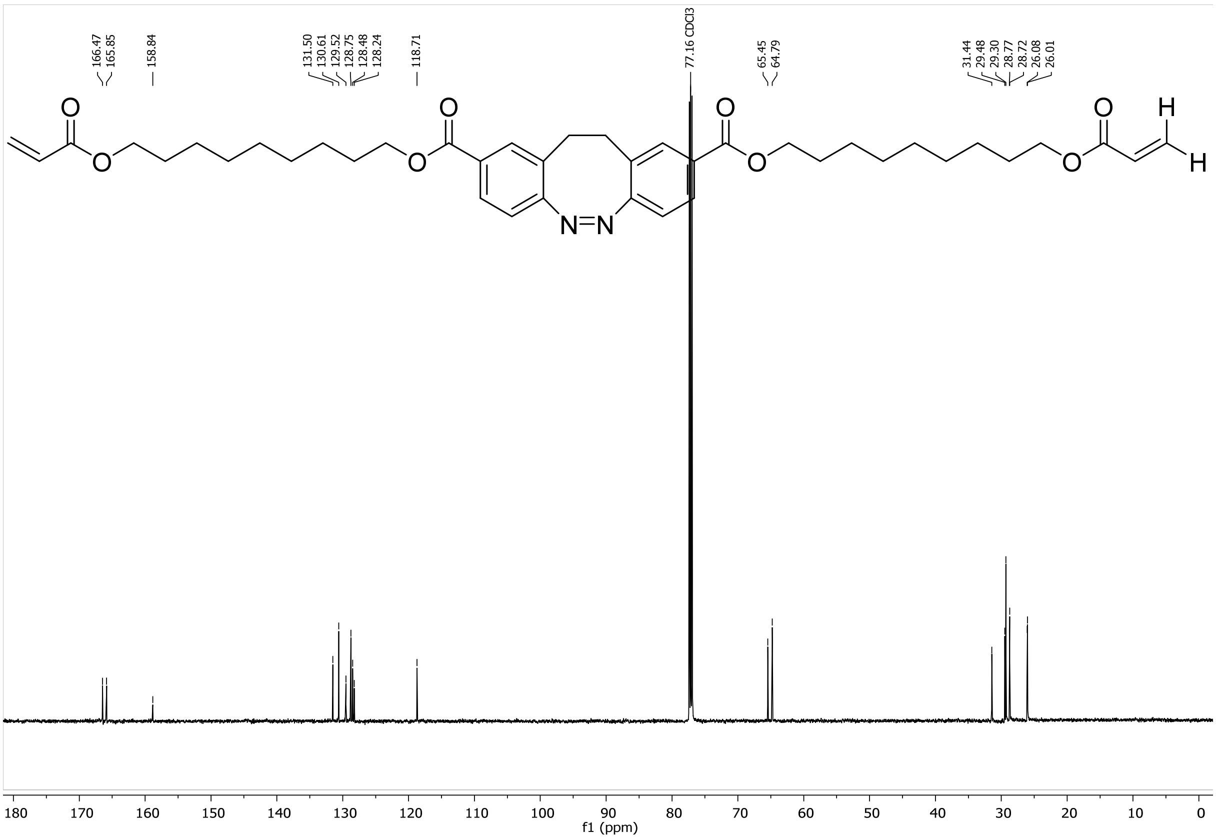

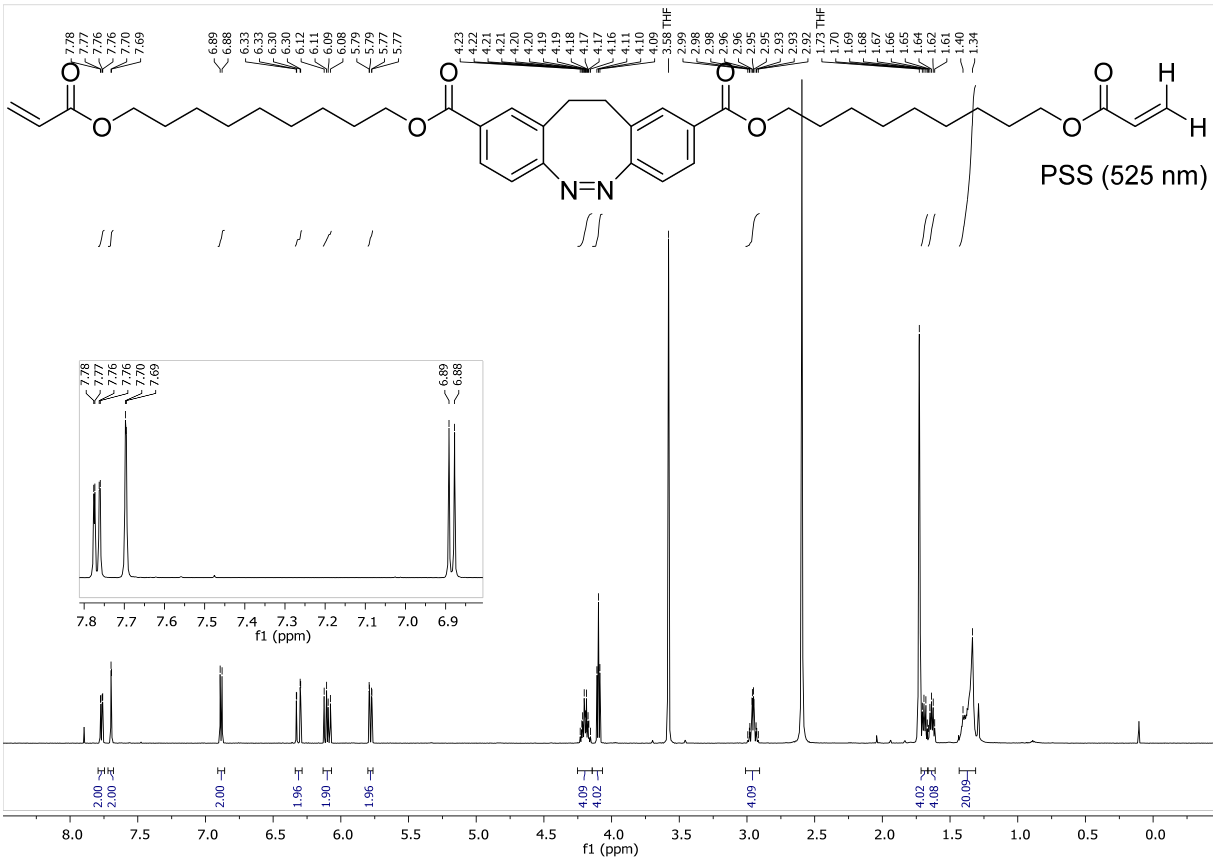
PSS (525 nm)
f1 (ppm)

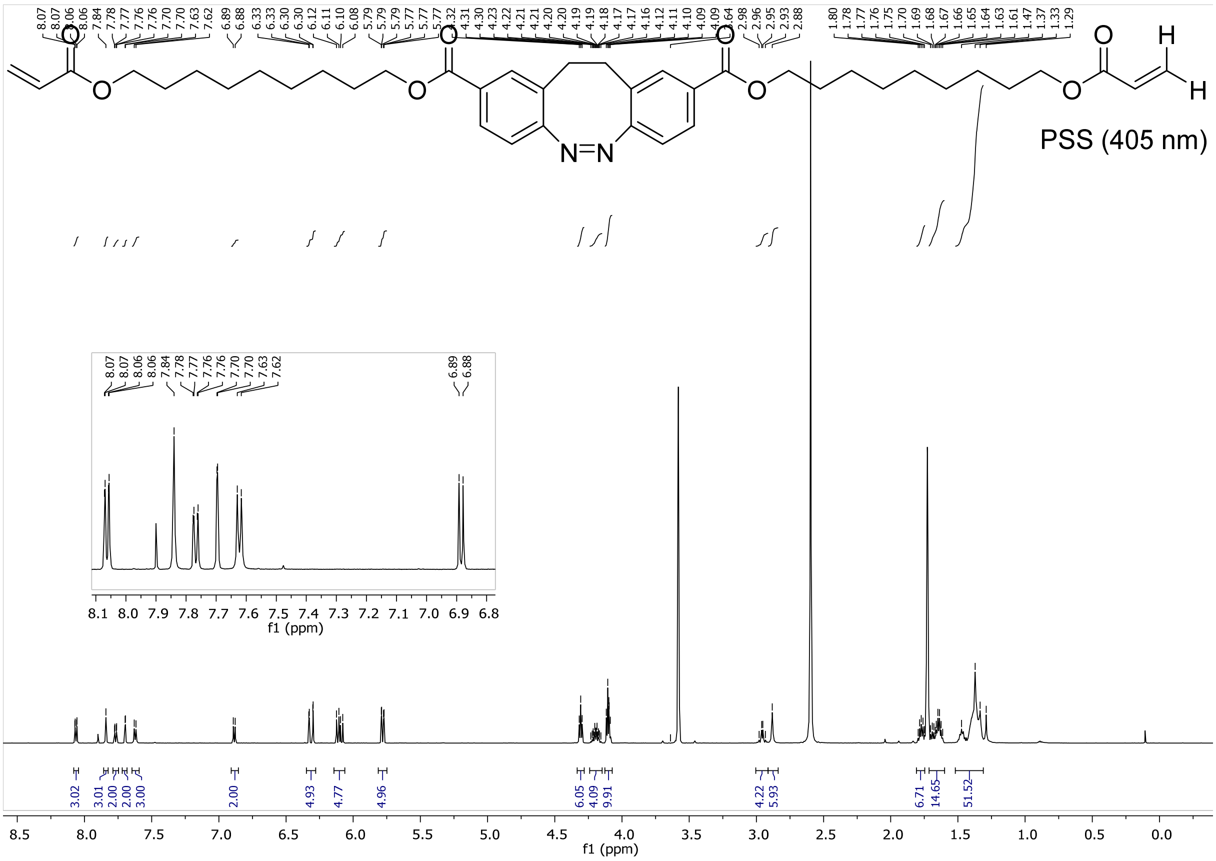
PSS (405 nm)
f1 (ppm)

(*Z*)-(11,12-Dihydrodibenzo[*c*,*g*][1,2]diazocine-2,9-diyl)bis(methylene) diacrylate (M2)

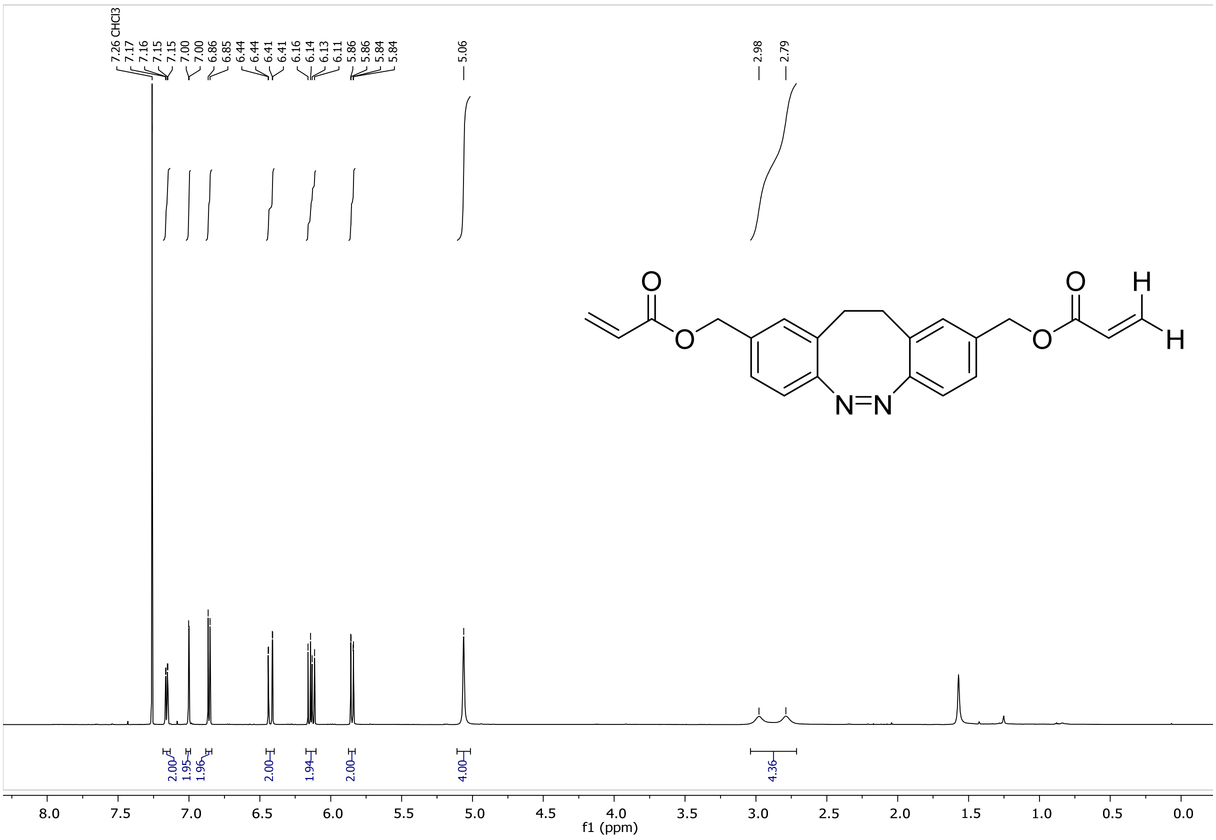

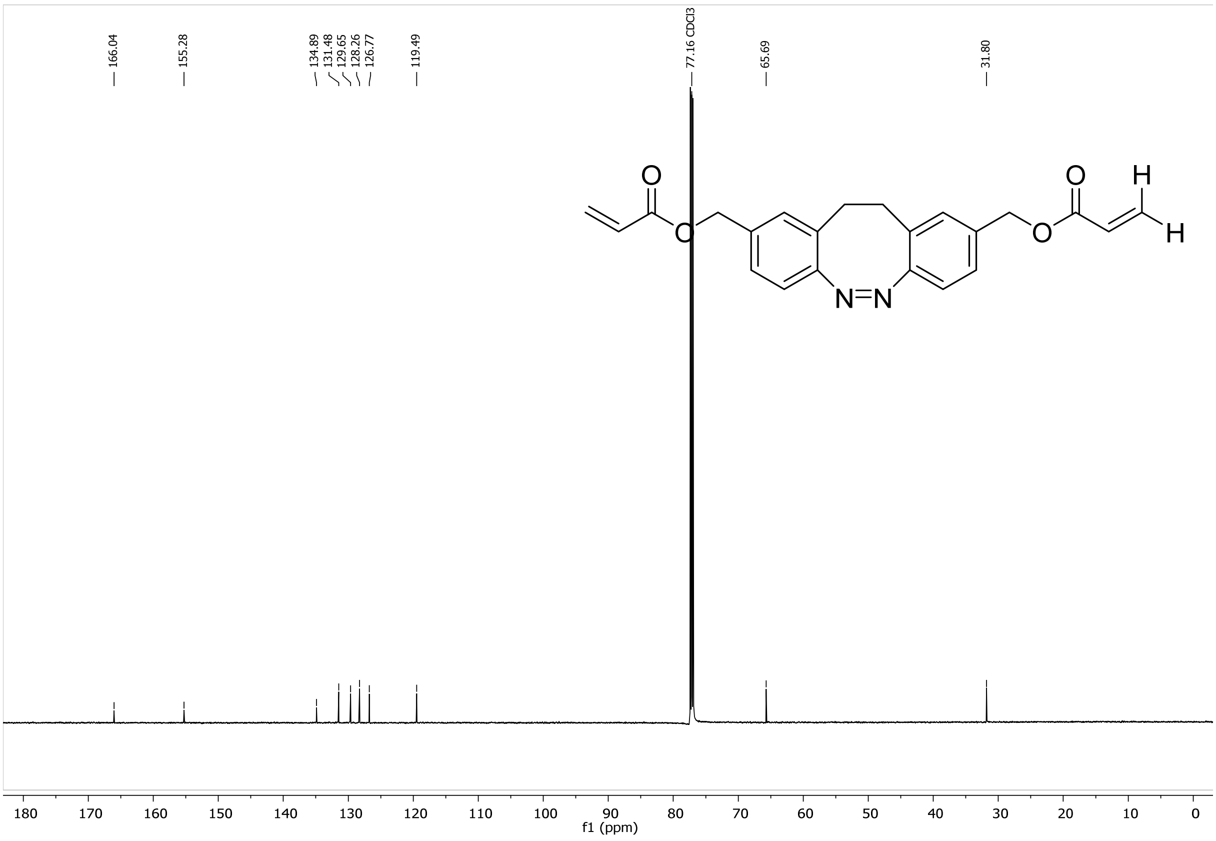

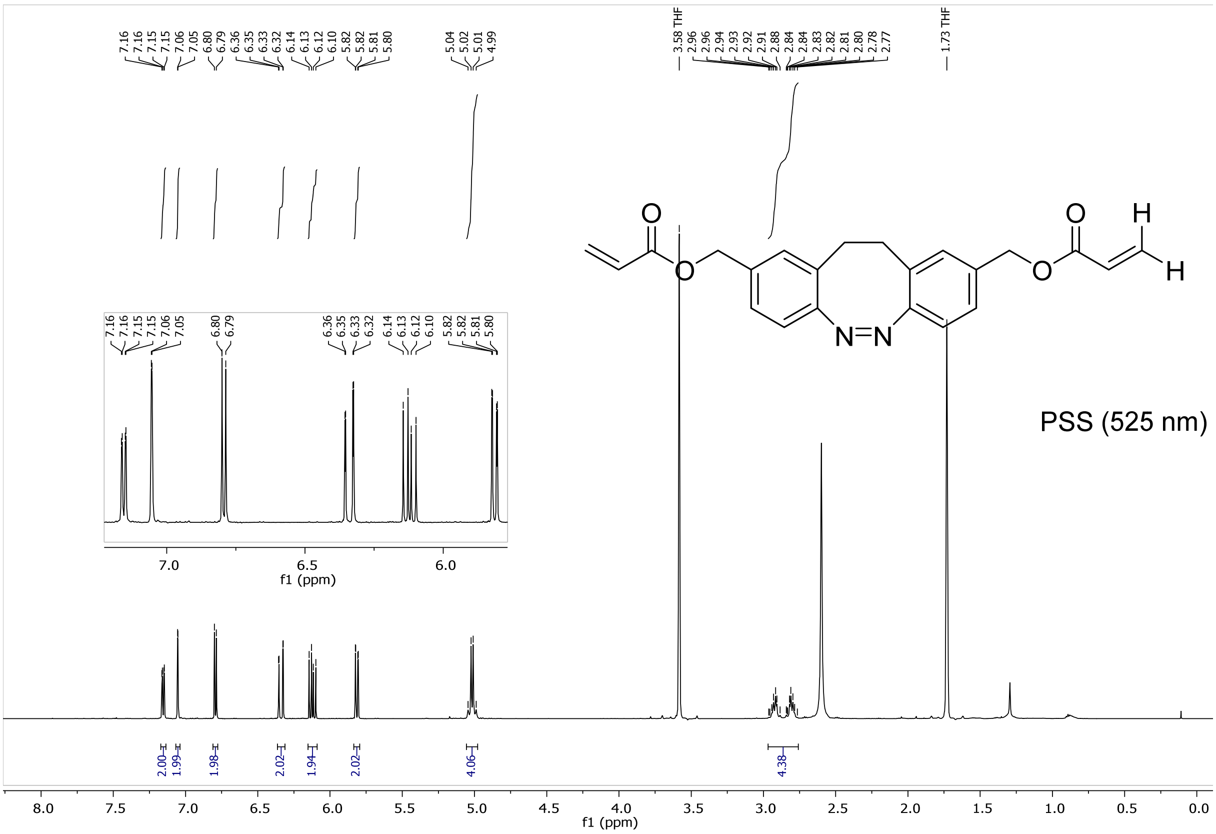
PSS (525 nm)
f1 (ppm)
3.58 THF
1.73 THF
N=N
O
H

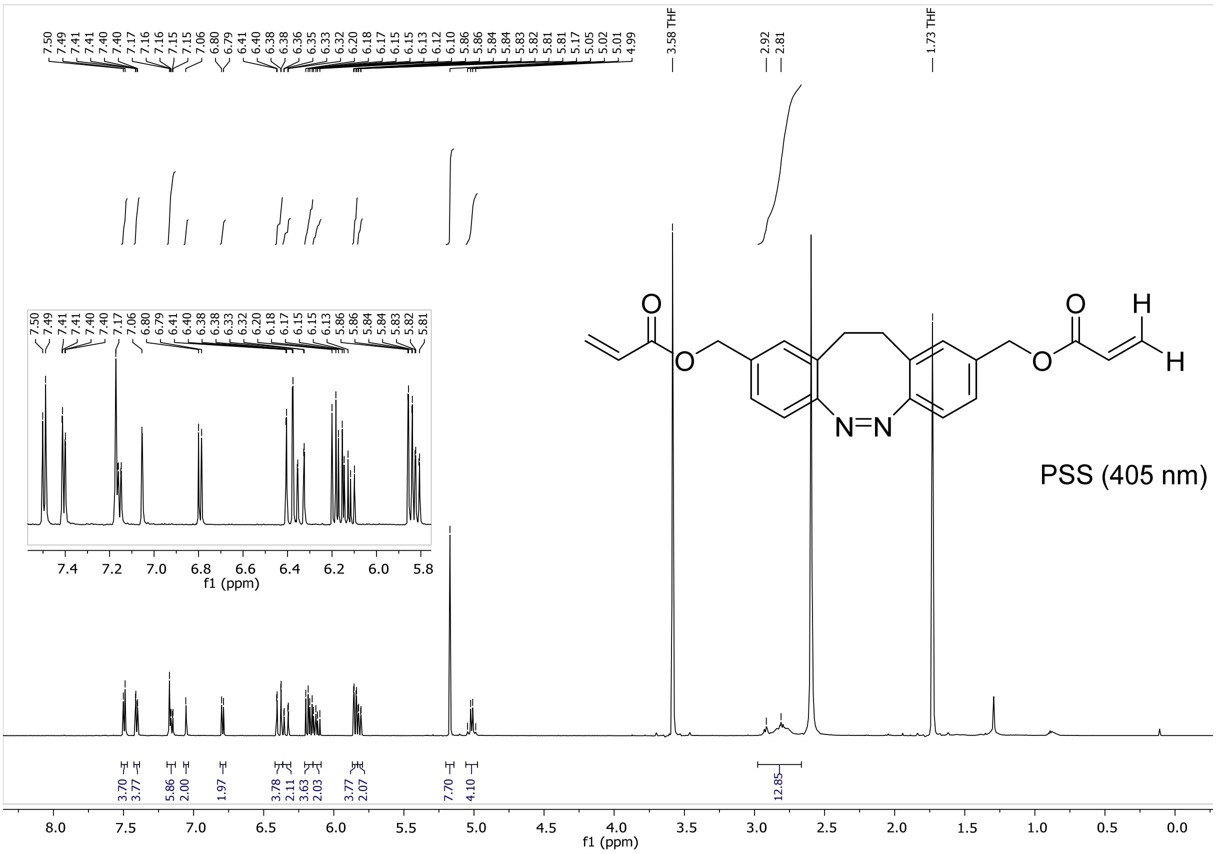
PSS (405 nm)
f1 (ppm)
3.58 THF
1.73 THF
N=N
O
H

Poly[3,3′-hexane-1,6-diylbis(sulfanediyl) bis(propionyloxynonyl) (*Z*)-(11,12-dihydrodibenzo[*c*,*g*][1,2]diazocine-2,9-diacarboxylate] (P1)

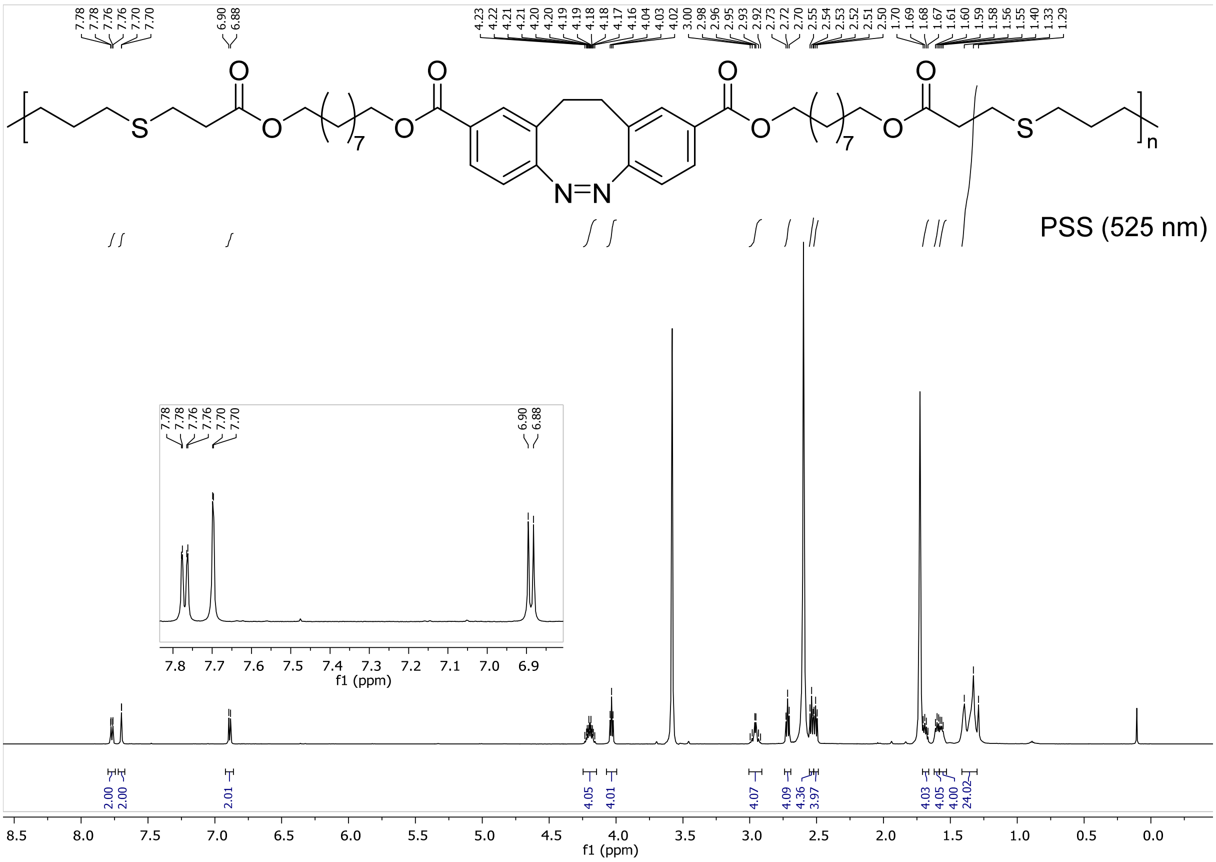

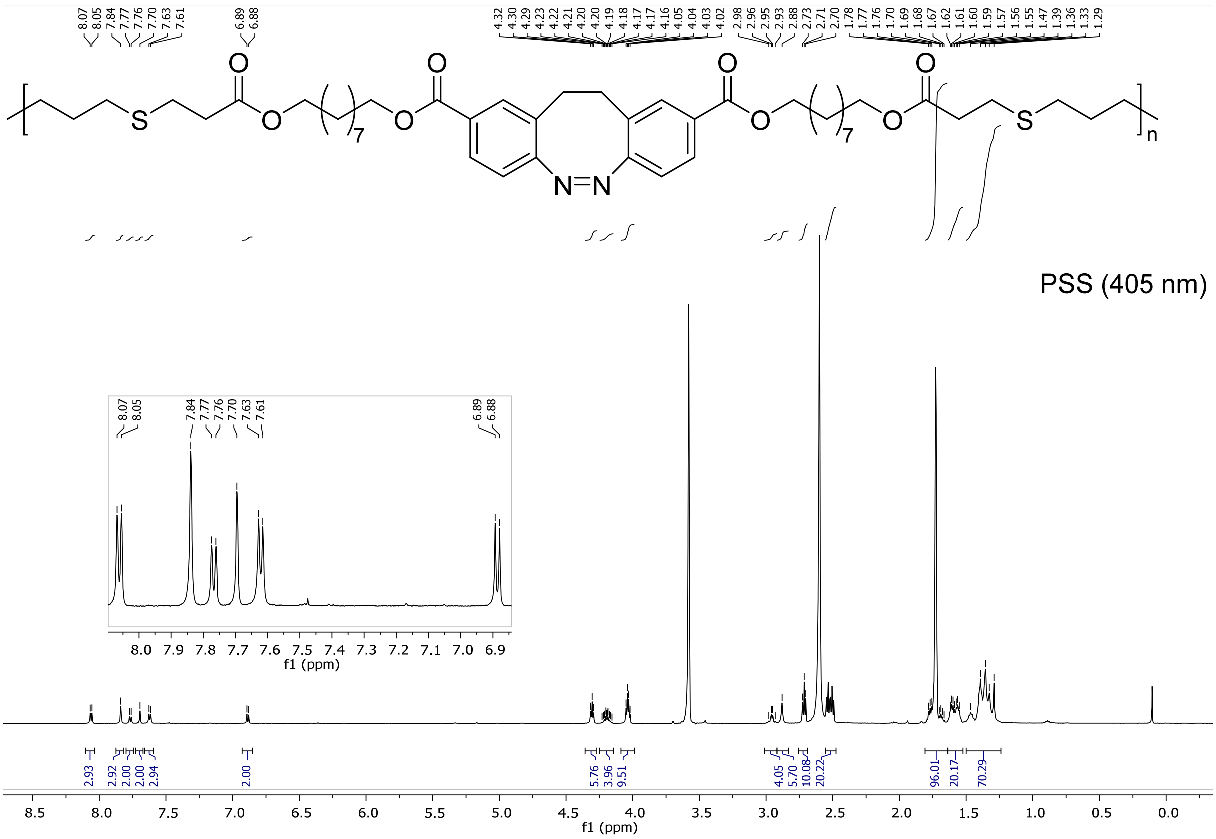

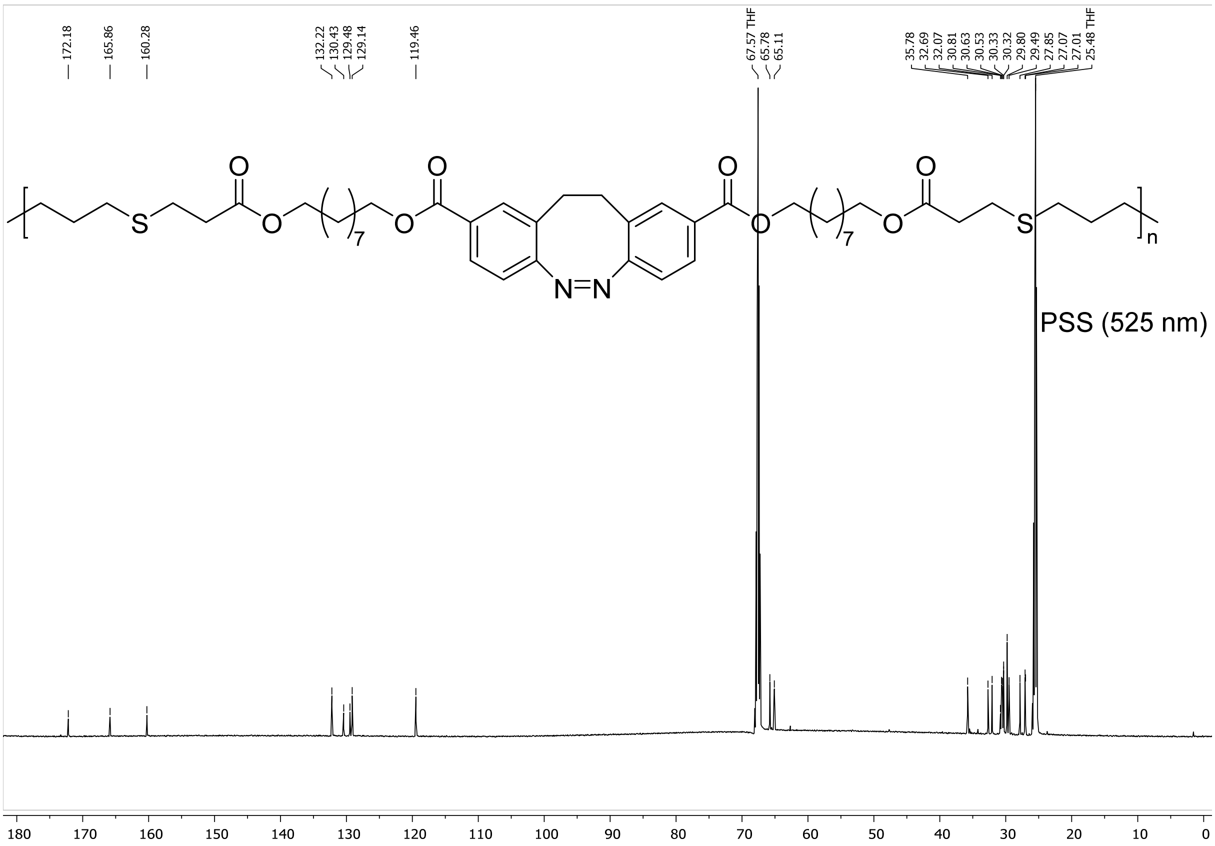
172.18
165.86
160.28
132.22
130.43
129.48
129.14
119.46
67.57 THF
65.78
65.11
35.78
32.69
32.07
30.81
30.63
30.53
30.33
30.32
29.80
29.49
27.85
27.07
27.01
25.48 THF
PSS (525 nm)
180 170 160 150 140 130 120 110 100 90 80 70 60 50 40 30 20 10 0

Poly[(Z)-(11,12-dihydrodibenzo[c,g][1,2]diazocine-2,9-dimethyl-3,3′-(hexane-1,6-diylbis(sulfanediyl))dipropionate] (P2)

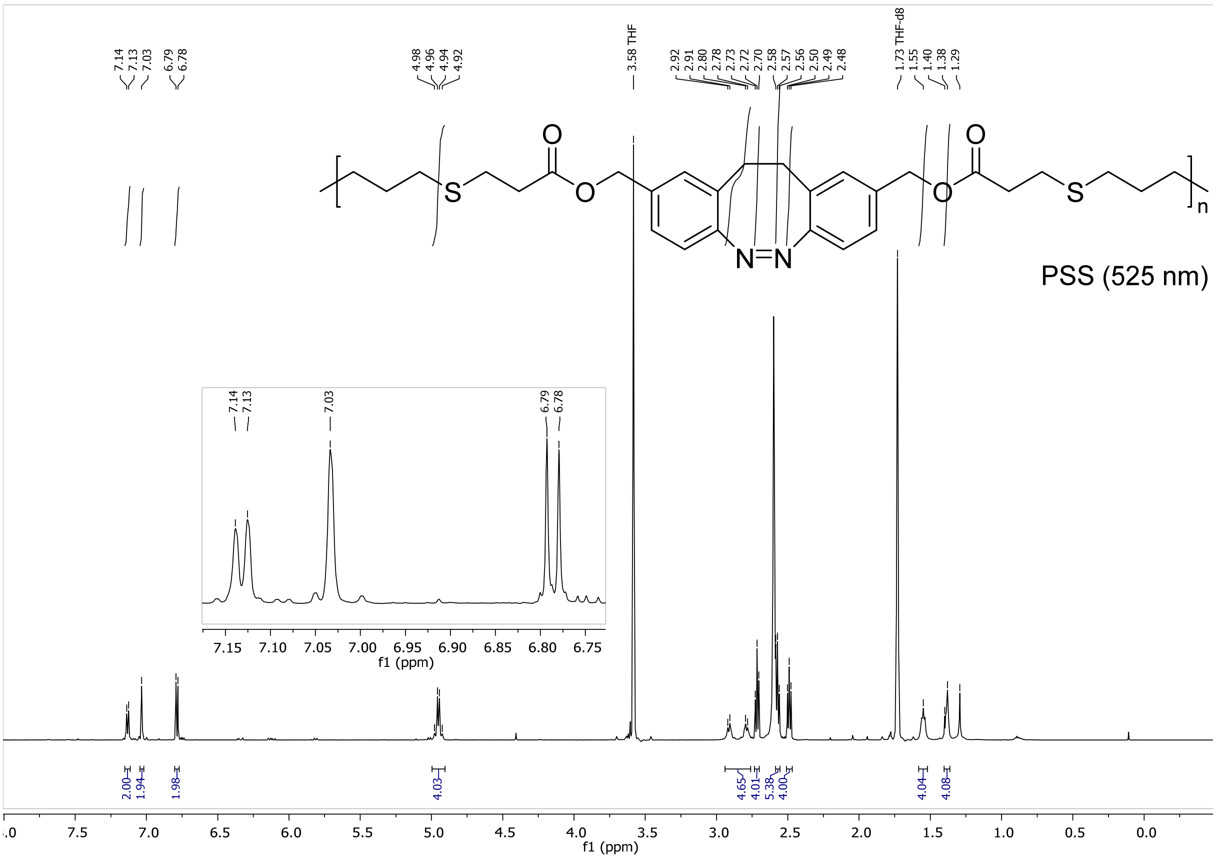

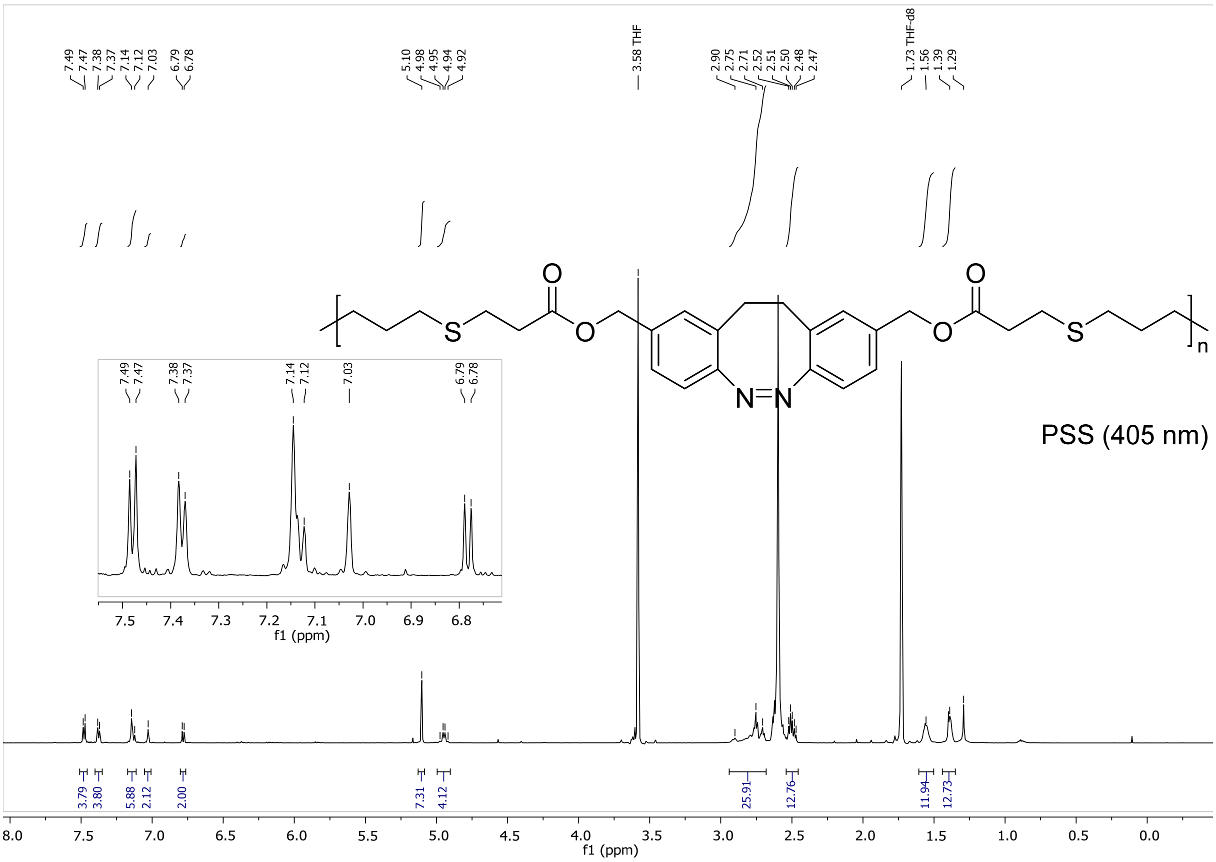
7.49
7.47
7.38
7.37
7.14
7.12
7.03
6.79
6.78
5.10
4.98
4.95
4.94
4.92
3.58 THF
2.90
2.75
2.71
2.52
2.51
2.50
2.48
2.47
1.73 THF-d8
1.56
1.39
1.29
O
S
O
N=N
n
PSS (405 nm)
3.79
3.80
5.88
2.12
2.00
7.31
4.12
25.91
12.76
11.94
12.73
f1 (ppm)

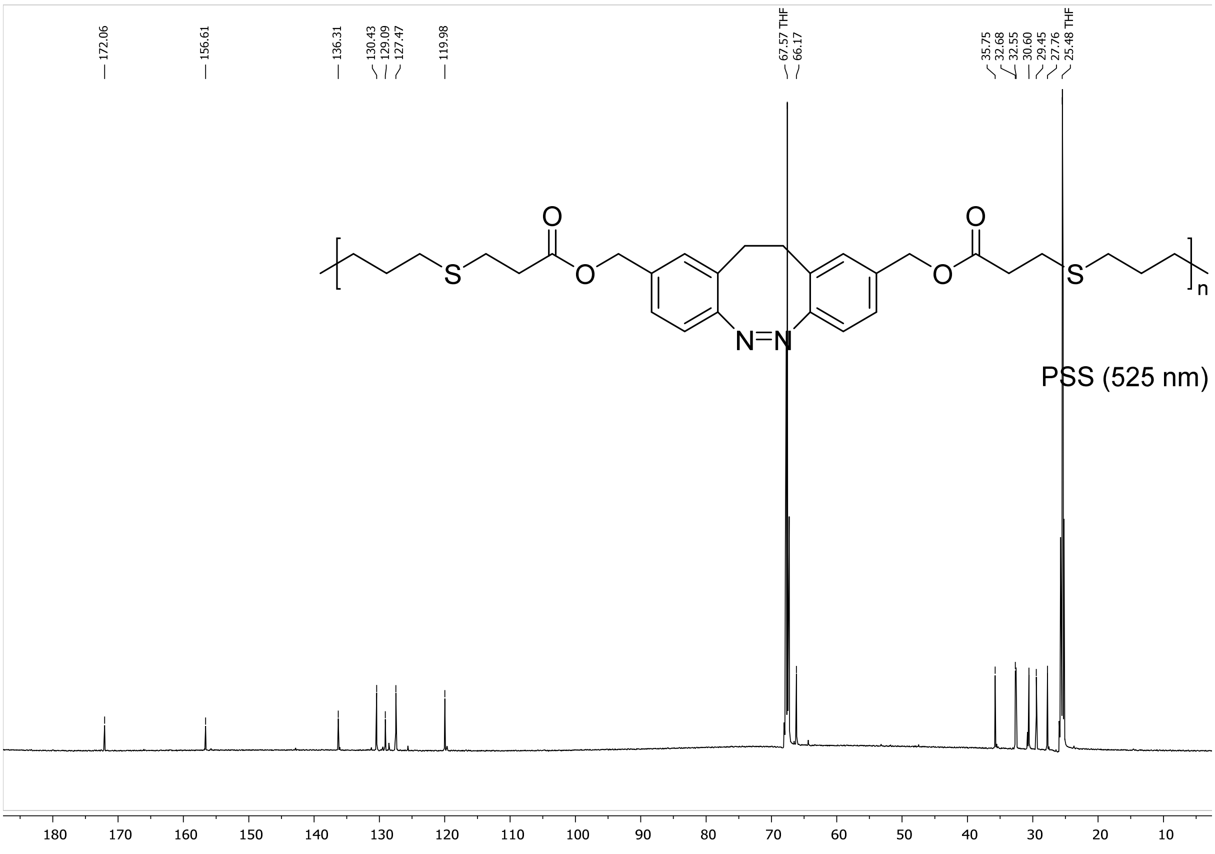
172.06
156.61
136.31
130.43
129.09
127.47
119.98
67.57 THF
66.17
35.75
32.68
32.55
30.60
29.45
27.76
25.48 THF
O
S
O
N=N
n
PSS (525 nm)

^{1}H DOSY NMR Spectra of M2 and P2

(*Z*)-(11,12-Dihydrodibenzo[*c*,*g*][1,2]diazocine-2,9-diyl)bis(methylene) diacrylate (M2)

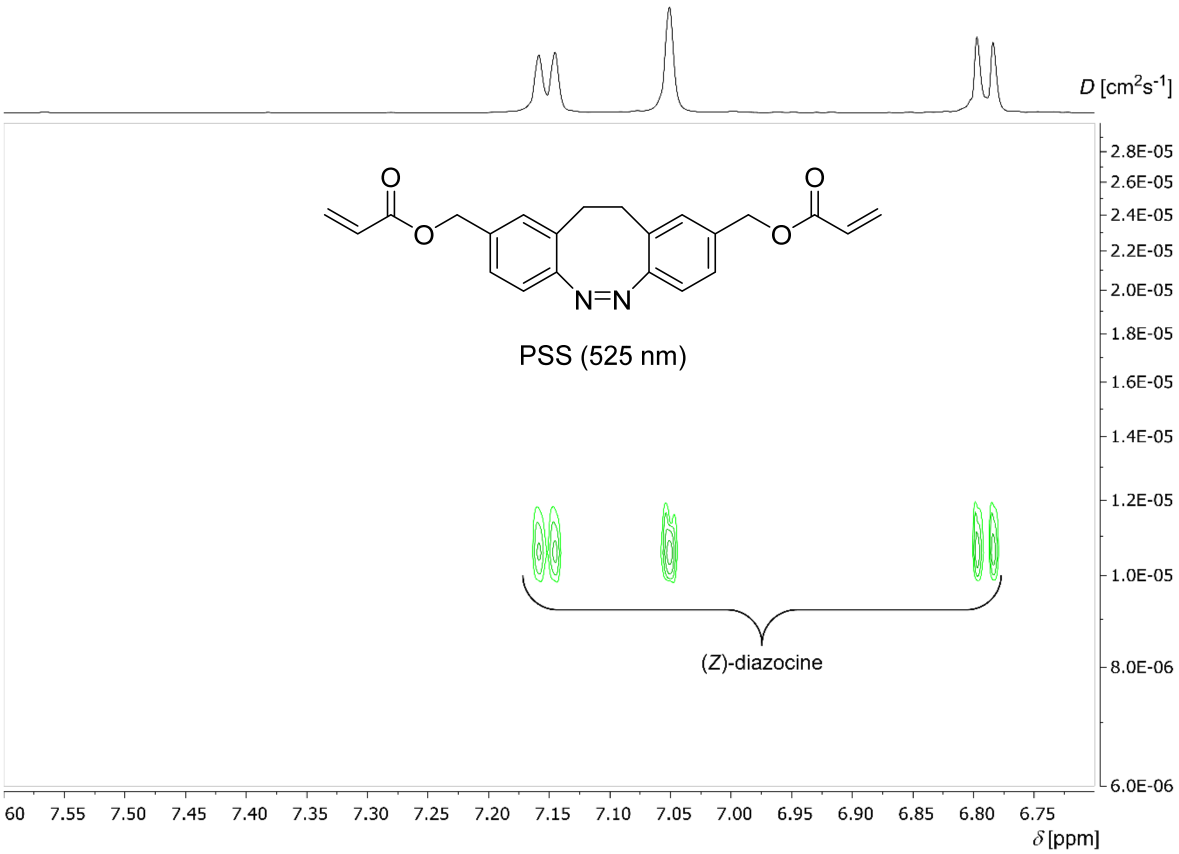

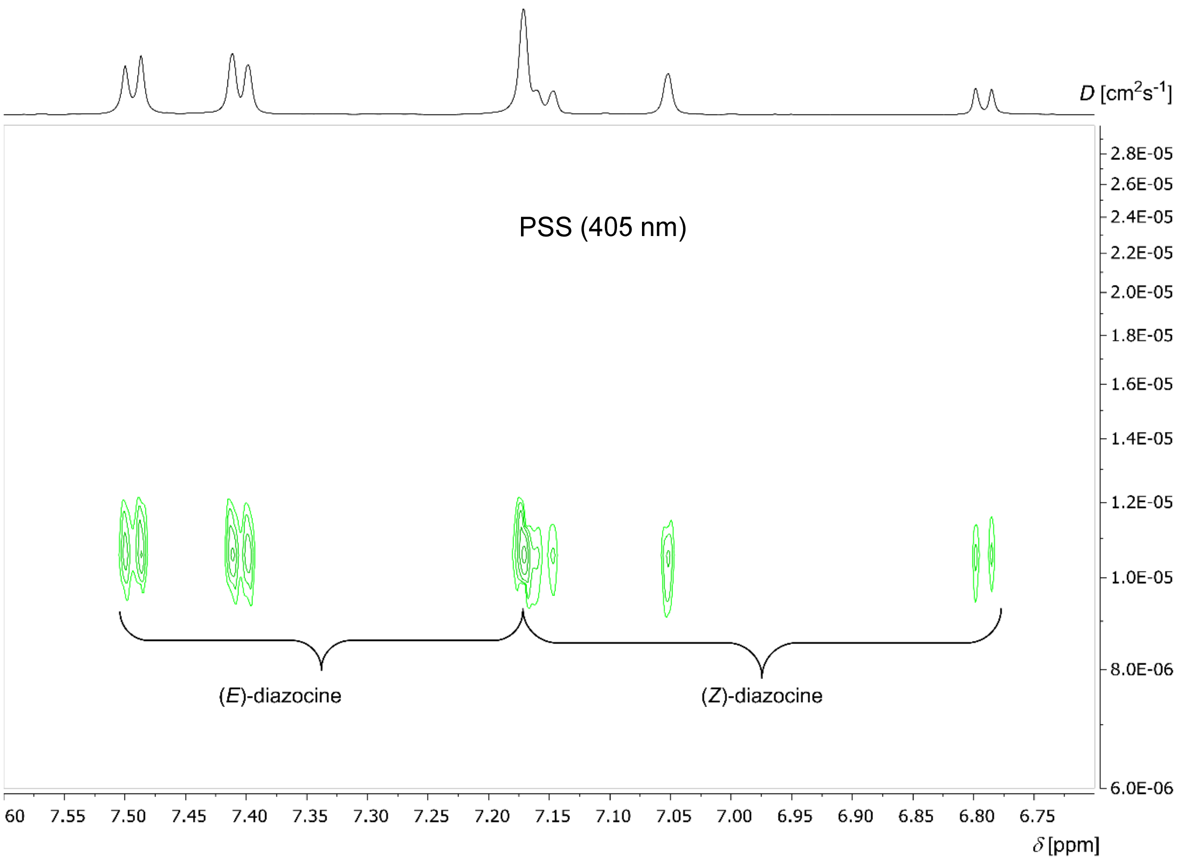

Poly[(*Z*)-(11,12-dihydrodibenzo[*c*,*g*][1,2]diazocine-2,9-dimethyl-3,3′-(hexane-1,6-diylbis(sulfanediyl))dipropionate] (P2)

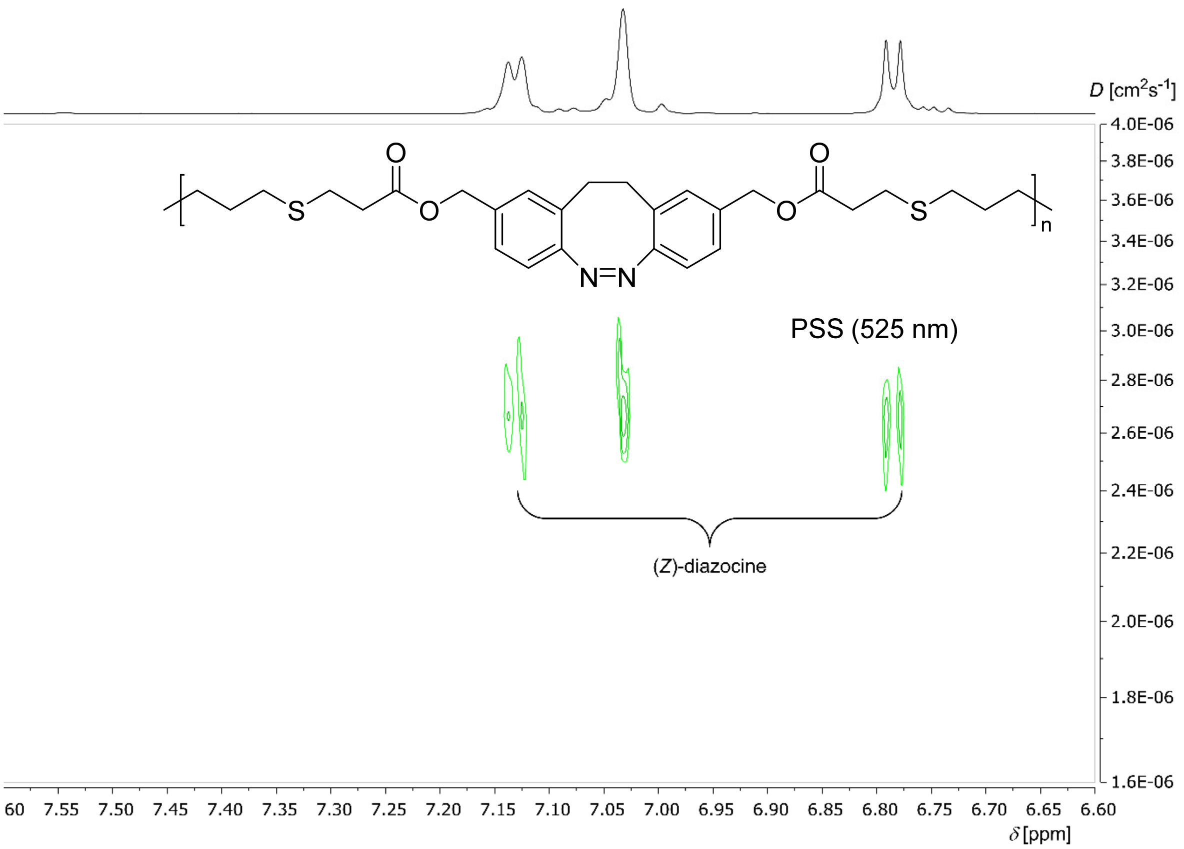

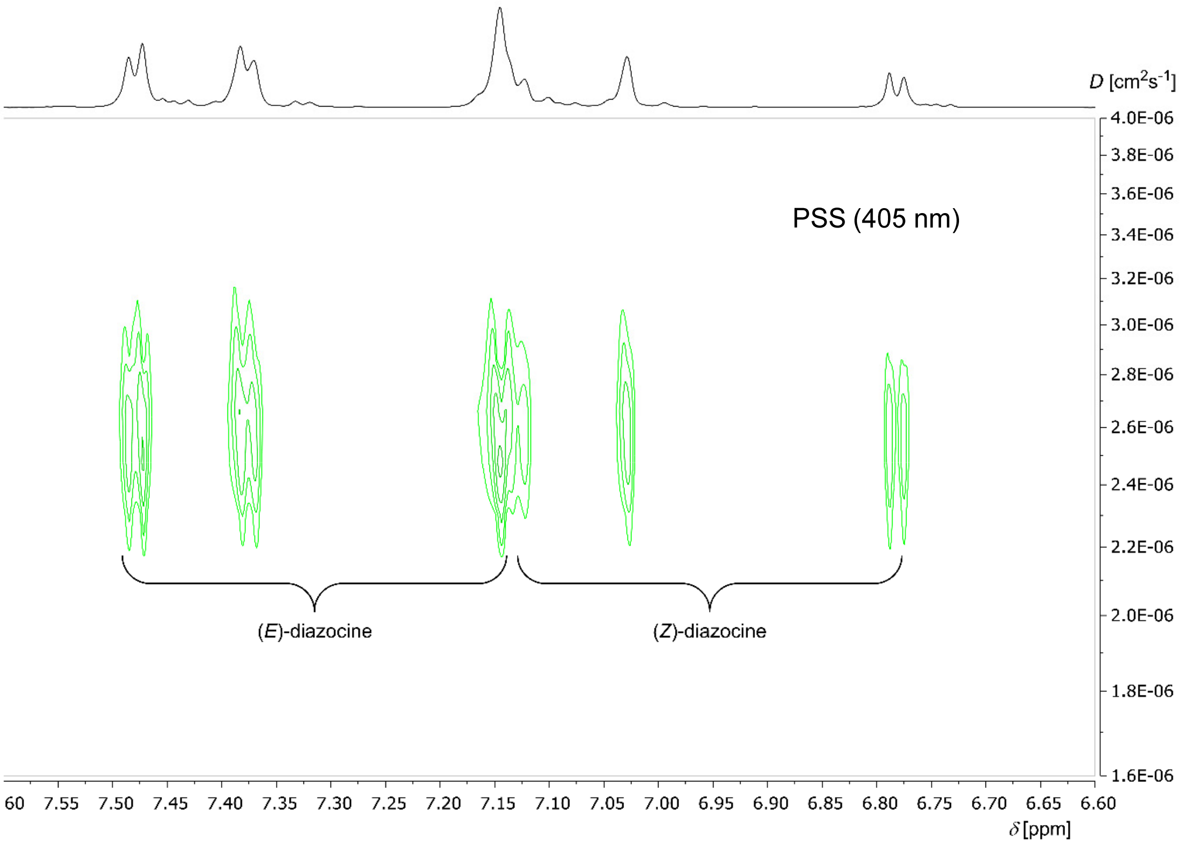

Supplement: Supplementary file 1 [file polymers-15-01306-s001.zip › polymers-2210150-supplementary.pdf]
